# Drosha Promotes Splicing of a Pre-microRNA-like Alternative Exon

**DOI:** 10.1371/journal.pgen.1004312

**Published:** 2014-05-01

**Authors:** Mallory A. Havens, Ashley A. Reich, Michelle L. Hastings

**Affiliations:** 1 Department of Cell Biology and Anatomy, Chicago Medical School, Rosalind Franklin University of Medicine and Science, North Chicago, Illinois, United States of America; 2 School of Graduate and Postdoctoral Studies, Rosalind Franklin University of Medicine and Science, North Chicago, Illinois, United States of America; 3 Department of Biology, Lake Forest College, Lake Forest, Illinois, United States of America; MRC Human Genetics Unit, United Kingdom

## Abstract

The ribonuclease III enzyme Drosha has a central role in the biogenesis of microRNA (miRNA) by binding and cleaving hairpin structures in primary RNA transcripts into precursor miRNAs (pre-miRNAs). Many miRNA genes are located within protein-coding host genes and cleaved by Drosha in a manner that is coincident with splicing of introns by the spliceosome. The close proximity of splicing and pre-miRNA biogenesis suggests a potential for co-regulation of miRNA and host gene expression, though this relationship is not completely understood. Here, we describe a cleavage-independent role for Drosha in the splicing of an exon that has a predicted hairpin structure resembling a Drosha substrate. We find that Drosha can cleave the alternatively spliced exon 5 of the *eIF4H* gene into a pre-miRNA both *in vitro* and in cells. However, the primary role of Drosha in *eIF4H* gene expression is to promote the splicing of exon 5. Drosha binds to the exon and enhances splicing in a manner that depends on RNA structure but not on cleavage by Drosha. We conclude that Drosha can function like a splicing enhancer and promote exon inclusion. Our results reveal a new mechanism of alternative splicing regulation involving a cleavage-independent role for Drosha in splicing.

## Author Summary

MicroRNAs (miRNAs) are short non-coding RNAs that function in gene silencing and are produced by cleavage from a larger primary RNA transcript through a reaction that is carried out by the Microprocessor. Primary miRNA transcripts are often located within the introns of genes. Thus, both the Microprocessor and the spliceosome, which is responsible for pre-mRNA splicing, interact with the same sequences, though little is known about how these two processes influence each other. In this study, we discovered that the alternatively spliced *eIF4H* exon 5 is predicted to form an RNA hairpin that resembles a Microprocessor substrate. We found that the Microprocessor can bind and cleave exon 5, which precludes inclusion of the exon in the mRNA. However, we find that Drosha, a component of the Microprocessor, primarily functions to enhance exon 5 splicing both *in vitro* and in cells, rather than to cleave the RNA. Our results suggest that the Microprocessor has a role in splicing that is distinct from its role in miRNA biogenesis. This Microprocessor activity represents a new function for the complex that may be an important mechanism for regulating alternative splicing.

## Introduction

MicroRNAs (miRNAs) are short non-coding RNAs that mediate posttranscriptional gene silencing by base-pairing with target mRNAs. The production of mature miRNAs occurs through a series of processing steps beginning with cleavage from the primary RNA transcript by the Microprocessor complex, which is minimally composed of the ribonuclease (RNase) III enzyme Drosha and the RNA binding protein DGCR8 [1]–[6]. Microprocessor cleavage generates a pre-miRNA of ∼60–70 nts which is subsequently exported to the cytoplasm and cleaved by a second RNase III enzyme, Dicer, into a mature miRNA that can target mRNAs [7]–[11].

Cleavage of an RNA transcript by Drosha in the Microprocessor complex occurs cotranscriptionally. Many miRNAs are intragenic, residing within introns and exons of other genes, and are transcribed from the host gene promoter [12], [13]. Splicing, the process by which non-coding introns are removed and exons are ligated together by the macromolecular spliceosome complex, also occurs cotranscriptionally. Thus, processing of intragenic miRNAs by the Microprocessor must occur in the context of splicing. The mechanisms that dictate the coordination of splicing and miRNA processing are not clearly understood.

Splicing and miRNA processing not only occur on shared RNA transcripts, but the proteins that are involved in these reactions are physically and functionally associated [2], [14]–[21], suggesting that splicing and pre-miRNA biogenesis may be more intimately associated than currently appreciated. Although studies have demonstrated that miRNA cleavage from introns does not affect splicing [13], [22], the influence of the two processes on each other is likely context-dependent. For example, the presence of exons flanking intronic miRNAs can promote miRNA production [23]. Likewise, excision of an intronic pre-miRNA has been shown to promote splicing, and splicing of an upstream exon, in turn, can promote pre-miRNA cleavage [24]. In contrast to these apparently cooperative activities, miRNA biogenesis and splicing are mutually exclusive when miRNAs are located in exons [25]–[27] or overlap splice sites [28], [29]. In addition, binding of the Microprocessor to alternative exons has been shown to limit mRNA isoform abundance [30], [31], further suggesting competition between splicing and miRNA biogenesis when the spliceosome and the Microprocessor recognize and cleave the same sequences.

Although the RNase III activity of Drosha in miRNA biogenesis is the most well documented function of Drosha, there is evidence that Drosha may also perform cleavage-independent activities in the nucleus. For example, in human cells, Drosha has been found in a larger protein complex comprised of proteins with unknown functions in miRNA biogenesis including several splicing factors [2]. This large complex cleaves RNA inefficiently *in vitro* and thus has the potential to have an alternative, cleavage-independent function in the cell [2]. In support of this notion, Drosha was recently shown to regulate the transcription of some genes in a cleavage-independent manner [32]. Thus, there appears to be contexts in which Drosha may function to influence gene expression in a manner that is not associated with miRNA biogenesis.

Here, we report on a new function for Drosha as an alternative splicing factor that enhances inclusion of an exon that is a Drosha-binding substrate. We find that the alternatively spliced exon 5 from the eukaryotic translation initiation factor 4H (*eIF4H*) gene transcript forms a hairpin structure that can be bound by the Microprocessor. We demonstrate that Drosha can cleave this exon in a canonical manner via the Microprocessor. However, Drosha expression does not cause a decrease in *eIF4H* mRNA abundance or lead to an increase in exon 5 skipping, which would be expected if cleavage were occurring efficiently. Rather, the predominant function of Drosha in *eIF4H* expression is to increase exon 5 splicing. This Drosha-mediated enhancement of splicing depends on the hairpin structure of the exon both in cells and *in vitro*. Our study identifies a mechanism for the regulation of splicing and miRNA biogenesis that involves a previously unappreciated role for Drosha in alternative splicing whereby Drosha enhances splicing of a regulated exon that resembles a canonical Drosha substrate.

## Results

### *eIF4H* exon 5 has a predicted hairpin structure resembling a Microprocessor substrate

**Figure 1:**
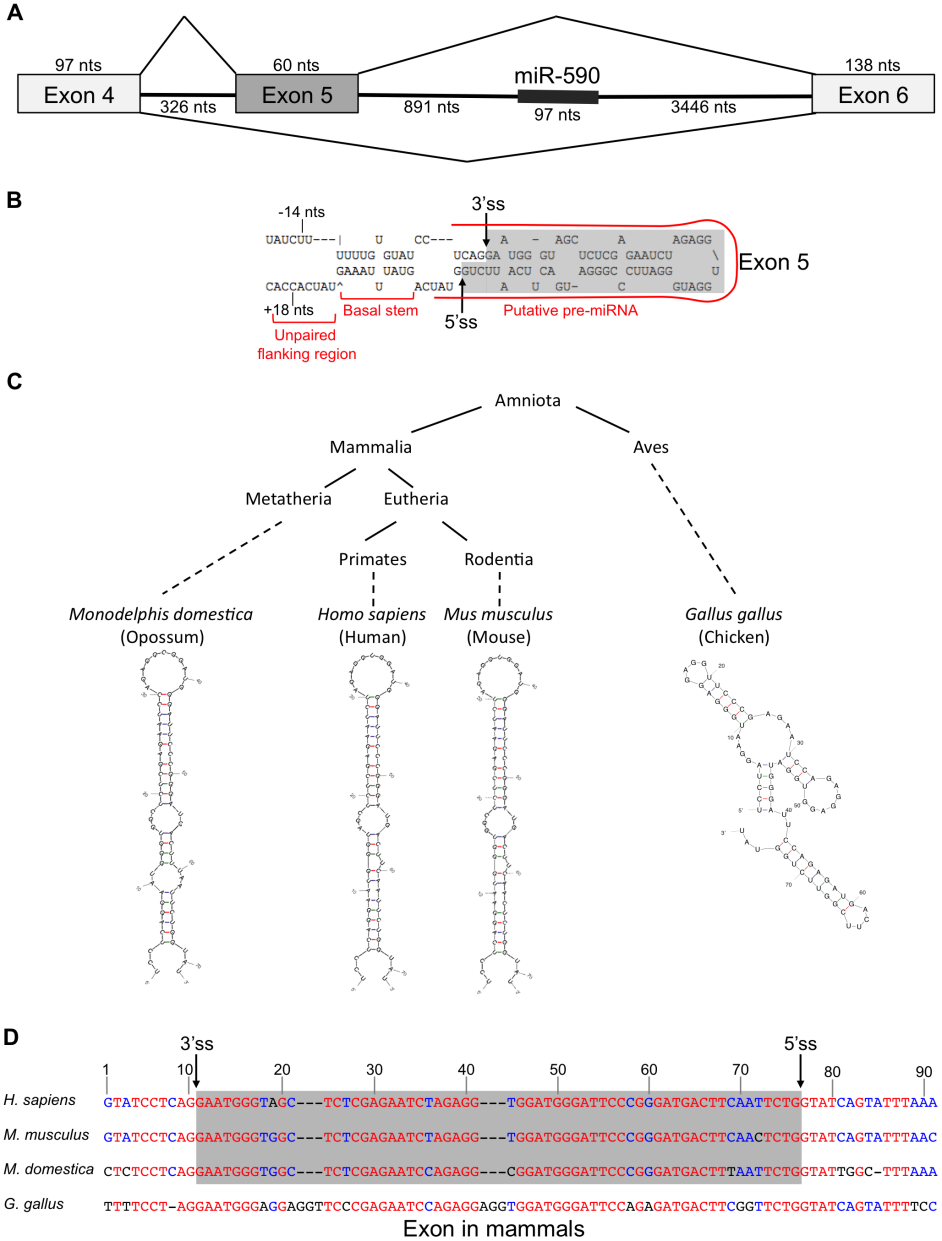
*eIF4h* exon 5 structure and conservation. (A) Organization of the *eIF4H* gene region from exon 4 to 6. Boxes represent exons and horizontal lines are introns. Diagonal lines show alternative splicing pathways. The thick line within intron 5 represents miR-590. The number of nucleotides (nts) for each region of the gene is included. (B) The sequence and predicted structure of human *eIF4H* exon 5 (shaded in gray) and flanking intronic sequences are shown. The putative pre-miRNA sequence is outlined with a red line. Sequences reported to promote Microprocessor substrate recognition and specificity [34] are noted including the upstream U at position −14 nts and a potential SRSF3 binding site (CNNC) at position +18 nts relative to the exon 5 splice sites. The 5′ (5′ss) and 3′ (3′ss) splice sites are shown with arrows. (C) A simplified phylogenetic tree depicting the conservation of exon 5 in several diverse species (above) and the corresponding predicted folded sequences of the exonic regions (below). (D) Sequence conservation of exon 5 and the flanking intronic regions. Red indicates nucleotide conservation across all four species. Blue indicates conservation in two or more species. Black indicates that the nucleotide is not conserved. Dashes represent gaps. The gray box refers to the exonic sequences.

In the course of studying the relationship between miR-590 processing and the splicing of the upstream alternative exon 5 of the *eIF4H* gene transcript, we discovered that exon 5, itself, is predicted to form a pre-miRNA-like hairpin structure (Figure 1A). Exon 5 is 60 nucleotides (nts) long, a length typical for a pre-miRNA [5]. When folded with flanking intronic sequences, the exon forms a hairpin with a basal stem of ∼11 base-pairs and unstructured nucleotides flanking the basal stem, all of which are features common to canonical miRNAs [33]. The exon also has sequence elements that have been predicted to contribute to Microprocessor substrate specificity [34], including a CNNC motif located 18 nts downstream (−18 nts) from a potential Microprocessor cleavage site [34], and a uridine positioned 14 nts upstream (+14 nts) from a putative Drosha cleavage site (Figure 1B) [34]. The sequence and structure of exon 5 are conserved in placental and non-placental (Opossum) mammals (Figure 1C and 1D). However, in chicken, the structure is not conserved and the sequence also is not recognized as an exon (Figure 1C and 1D), indicating a possible co-evolution of the exon and structure. Together, these results suggest that the structure may be important for splicing of this regulated exon, and raise the possibility that the Microprocessor interacts with this structure, which could have an effect on splicing.

### The Microprocessor can cleave *eIF4H* exon 5

**Figure 2:**
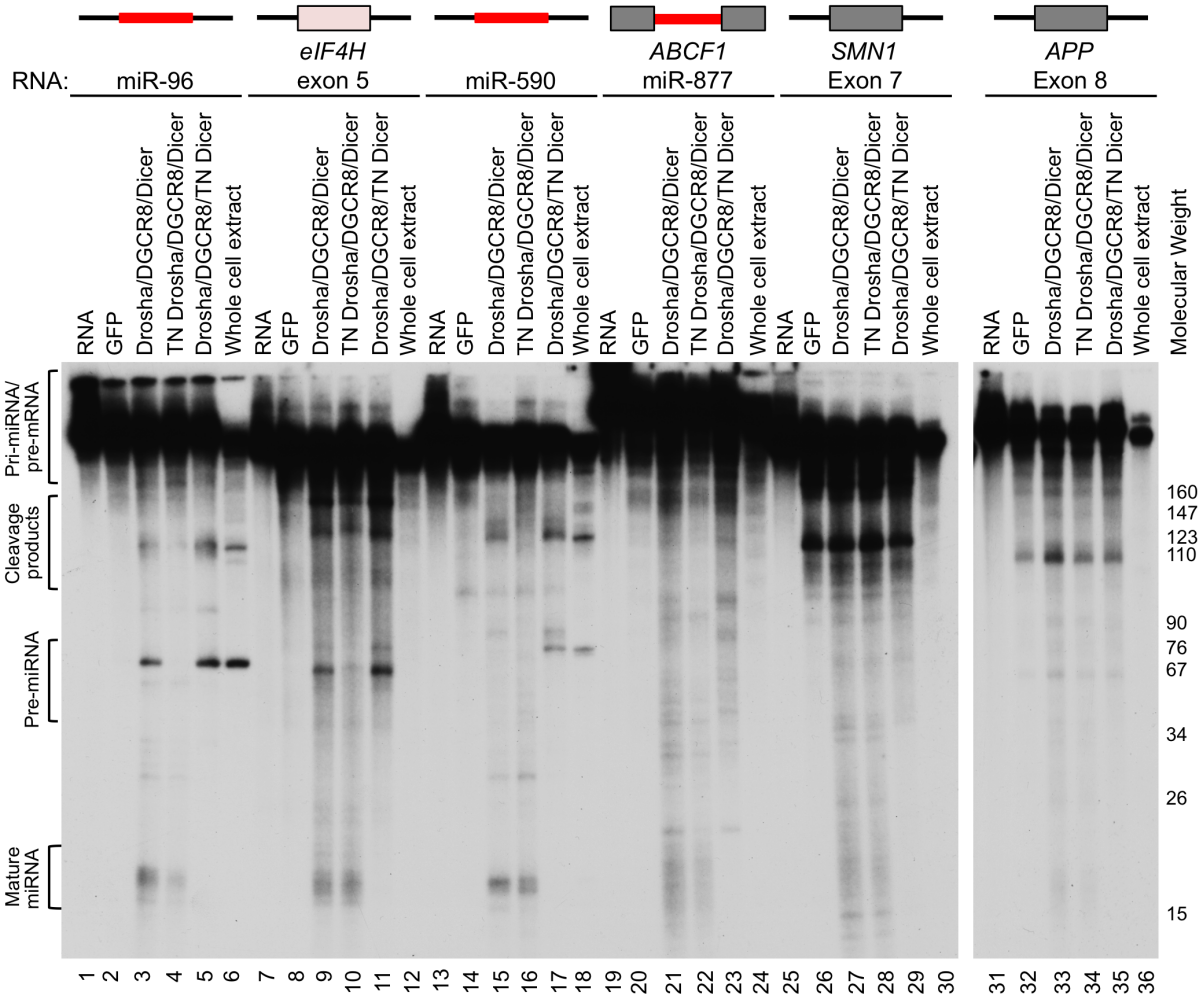
The Microprocessor and Dicer cleave *eIF4H* exon 5 *in vitro*. *In vitro* transcribed, radiolabelled *eIF4H* exon 5 or control RNAs were incubated with immunopurified FLAG-GFP (GFP), FLAG-Drosha or the transdominant negative version (TN Drosha), FLAG-DGCR8 and FLAG-Dicer or the transdominant negative version (TN Dicer), or whole cell extract. The canonical miRNAs, miR-96 (lanes 1–6) and miR-590 (lanes 13–18), are positive controls and the microprocessor-independent miRNA, mirtron miR-877 (lanes 19–24), located in host gene *ABCF1*, exon 7 from *SMN1* (lanes 25–30) and exon 8 from *APP* (lanes 31–36), are negative controls. *eIF4H* exon 5 is shown (lanes 7–12). Grey boxes indicate exons, black lines indicate introns, red lines indicate known miRNA sequences. Molecular weight marker sizes are indicated on the right. The different RNA species are labeled on the left. Template RNA was loaded as a size marker (RNA, lanes 1, 7, 13, 19, 25, 31).

To test whether *eIF4H* exon 5 can be processed into small RNAs by the miRNA processing machinery, we performed *in vitro* miRNA processing experiments. Exon 5 and the flanking intronic sequences were transcribed *in vitro* and incubated with immunopurified, FLAG-tagged Drosha, DGCR8 and Dicer, the RNase III enzyme that cleaves pre-miRNA to generate a mature ∼20–23 nt miRNA [8], [9]. Transdominant negative (TN) Drosha and TN Dicer, which can bind to but not cleave RNA substrates [35], were also tested. We found that the Microprocessor and Dicer cleaved *eIF4H* exon 5 into ∼60 and ∼20 nt fragments, sizes that are consistent with cleavage by Drosha and Dicer into a pre-miRNA and mature miRNA, respectively (Figure 2). Exon 5 processing by the Microprocessor was comparable to that of canonical miRNAs miR-96 and miR-590 (Figure 2). Other RNA substrates that are comprised of alternative exons, *APP* exon 8 and *SMN1* exon 7, are not predicted to form hairpins (Figure S1), and were not cleaved in a specific manner by the components of the miRNA biogenesis pathway (Figure 2). Additionally, the Microprocessor did not arbitrarily process hairpin RNAs, as it did not cleave miR-877, a mirtron with a hairpin structure that relies on splicing rather than the Microprocessor for excision from the primary RNA transcript [36]–[38] (Figure 2). The transdominant negative versions of Drosha and Dicer did not cleave *eIF4H* exon 5 into pre- or mature miRNA, respectively, indicating that exon 5 excision from the RNA transcript requires the RNase activity of these proteins (Figure 2). These results demonstrate that the Microprocessor and Dicer can specifically bind and cleave *eIF4H* exon 5. Whole cell extract did not efficiently process *eIF4H* exon 5 into a pre-miRNA, possibly indicating that other factors present in these complete extracts, and absent in the purified Microprocessor complex, may inhibit Drosha cleavage of exon 5.

**Figure 3:**
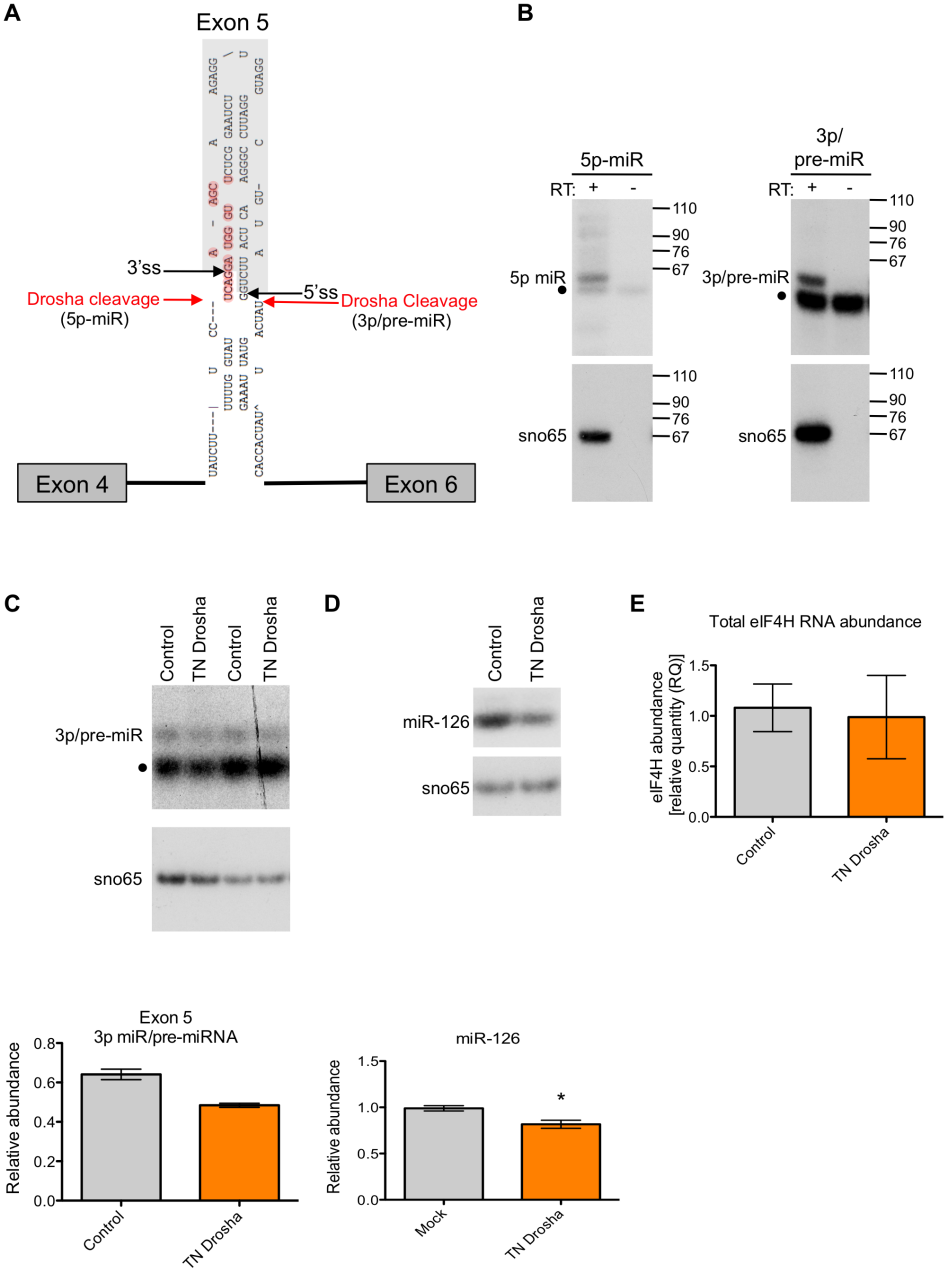
Exon 5 is cleaved from RNA *in vitro* and in cells. (A) RNA products isolated from a region of the gel from Figure 2 corresponding to RNAs migrating between 30–80 nts in length were subjected to 5′ and 3′ RACE. Red arrows indicate 5′ and 3′ ends that were identified through sequencing of cleaved RNA products that represent the 5′ end and 3′ ends of putative 5p and 3p miRNAs, respectively. Black arrows indicate the 5′ and 3′ splice sites (ss). Bases outlined in red indicate a putative Dicer cleavage product as predicted by PhDCleav [39]. The gray shading in the RNA structure represents exon 5. (B) Radiolabelled, stem-loop RT-PCR analysis of total cellular RNA, with (+) or without (−) reverse transcriptase, using stem-loop primers specific to the ends detected by sequencing of 3′ RACE products, or predicted by PHDCleav. SnoRNA65 (sno65) is a control, indicating the specific detection of RNA products. The stem-loop primer adds 38 nts to the amplicons, thus the 5p-miR and 3p/pre-miR are ∼36 nts and ∼27 nts, respectively, without the stem-loop. Molecular weight marker sizes are shown on the right. (C) Radiolabelled stem-loop RT-PCR analysis of exon 5 3p/pre-miR (n = 2) or (D) miR-126 (n = 3) in RNA isolated from HEK-293T control cells or cells transiently transfected with TN Drosha. Sno65 is a loading control. The graphs show the relative abundance of exon 5 3p/pre-miR and miR-126 RNA (miR/sno65). • indicates primer dimers. (E) *eIF4H* overall abundance as determined by Taqman qRT-PCR. Relative RNA quantity (RQ) was calculated using the ΔΔCt method where β-actin was the control, n = 6. In all cases, * indicates statistical significance determined by the Student's t-test where p≤0.05. Error bars represent standard error of the mean (SEM).

To determine the nature of the *eIF4H* exon 5 cleaved products, we isolated the small RNAs (30–80 nts) from the *in vitro* processing gel and identified the terminal ends by 5′ and 3′ rapid amplification of cDNA ends (RACE). This technique only allows the detection of RNA species containing a 5′-terminal phosphate and 3′-terminal hydroxyl group, which are indicative of specific RNA cleavage rather than hydrolysis or degradation. The Microprocessor generated several distinct 5′ and 3′ ends within the exon and adjacent to the splice sites in the flanking introns (Figures 3A and S2). We conclude from these results that cleavage of *eIF4H* exon 5 by the Microprocessor can effectively remove the exon from the pre-mRNA, thus demonstrating an interaction between exon 5 and the Microprocessor.

We next asked whether exon 5 is cleaved by the Microprocessor in cells. We carried out 5′ RACE on total RNA from HEK-293T cells to determine the sequence and termini of exon 5-derived, cleaved RNAs. We identified some of the same 5′ ends in cells that we found *in vitro* (Figure S2), which suggests that the exon 5 cleavage by the Microprocessor complex also occurs in cells.

To test whether mature miRNAs are generated from exon 5 in cells, we predicted miRNA sequences based on the identified Drosha cleavage sites and extrapolation of Dicer cleavage sites using the prediction algorithm, PHDcleav [39] (Figure 3A). We designed stem-loop primers for reverse transcription that were specific for all predicted mature miRNA 3′ ends and the 3′ ends detected by 3′RACE (Figure S2), and determined the actual cleavage sites based on the detection of a product following RT-PCR with the matching stem-loop primer (See Table S1 for stem-loop primers). We detected both a putative mature 5p-miRNA and a 3p/pre-miRNA generated from exon 5 in cells (Figure 3B), albeit at low abundance.

To confirm the presence of the exon 5 small RNAs in cells, we performed Northern blot analysis of total RNA from naïve HEK-293T cells or cells transfected with a minigene expressing an RNA that includes *eIF4H* exons 4 through 6 and the intervening intronic sequences. We analyzed the blots using probes specific for either exon 5 or miR-590, which is also expressed from the minigene and was included as a positive control. We detected a putative pre-miRNA species of ∼70 nts (Figure S3), which is ∼10 nts longer than the predominant cleavage product generated in the *in vitro* processing assay, which may indicate that cleavage in cells in the context of splicing occurs more frequently in the intronic sequences flanking exon 5, consistent with 5′ RACE results (Figure S2, product 10). We did not detect an appreciable amount of endogenous or minigene-derived mature or pre-miRNA species derived from exon 5 in cells (Figure S3), indicating that Drosha cleavage of exon 5 may be inefficient *in vivo*, similar to cleavage in whole cell extracts in the *in vitro* assay (Figure 2). We also detected both a pre-miR-590 and mature miR-590-5p in the *eIF4H* mini-gene transfected cells only, suggesting a low abundance of endogenous miR-590 in these cells (Figure S3).

We next tested whether the exon 5 3p-miRNA/pre-miRNA is a bona fide Drosha cleavage product. For this, we transfected HEK-293T cells with transdominant negative (TN) Drosha which binds RNA without cleaving, and also reduces endogenous Drosha cleavage by competitively binding to substrates [3], [35] (Figure 3). We found that the abundance of exon 3p/pre-miRNA and a control miRNA, miR-126, decreased when TN Drosha is expressed in cells (Figure 3C and 3D) as expected for a Drosha-dependent substrate. This result confirms that Drosha can cleave exon 5 in cells.

To investigate whether this decrease in cleavage of exon 5 had an effect on *eIF4H* expression, we analyzed total *eIF4H* abundance by real-time quantitative PCR with primers to a common region of the gene transcript. We found that TN Drosha expression does not result in an increase in the overall abundance of *eIF4H* that would be expected to accompany a decrease in cleavage of *eIF4H* exon 5 (Figure 3E). We conclude that either Drosha cleavage of exon 5 is such a rare event that it does not alter *eIF4H* abundance, or cleavage of the exon results in splicing from exon 4 to exon 6 in transcripts that have been cleaved by Drosha in exon 5, resulting in no net reduction in total *eIF4H* mRNA.

### Drosha enhances exon 5 splicing

**Figure 4:**
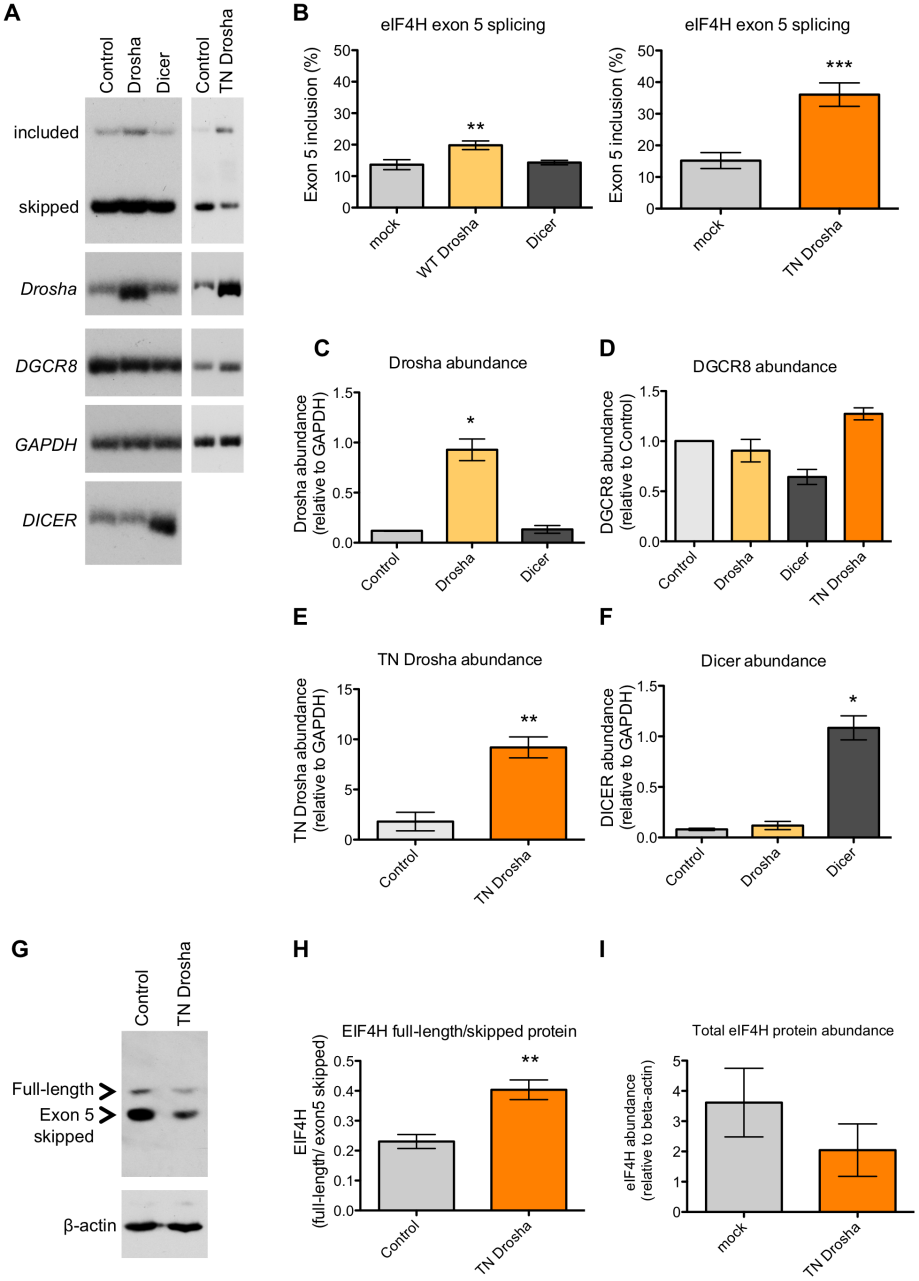
Drosha enhances exon 5 splicing. (A) Radiolabelled RT-PCR analysis of endogenous *eIF4H* splicing, and expression of *Drosha*, *DGCR8*, *DICER* and a loading control, *GAPDH*, in HEK-293T control cells or following transfection with Drosha, Dicer or TN Drosha. (B) Quantitation of exon 5 splicing. The graphs show the percent of exon 5 splicing [included/(included+skipped)*100]; TN Drosha n = 9; Drosha and Dicer n = 3. The lower graphs show quantitation of (C) *Drosha* (D) *DGCR8* (E) *TN Drosha* and (F) *DICER* abundance following overexpression, relative to *GAPDH*, n = 3. (G) Immunoblot of EIF4H protein isoform expression in the absence (control) or presence of TN Drosha expression in HEK-293T cells. (H) The graph shows the ratio of isoform expression (full length/skipped), n = 4. (I) The graph shows the overall protein expression of EIF4H [(Full Length+Skipped)/β-actin], n = 4. * p≤0.05, ** p≤0.005, *** p≤0.0005 indicate statistical significance by Student's t-test, All error bars represent SEM.

To determine whether Drosha-cleavage of exon 5 leads to a change in the alternative splicing of the exon, we overexpressed wild-type Drosha in HEK-293T cells and analyzed splicing by RT-PCR (Figure 4A, 4B and 4C). We predicted that Drosha overexpression would increase exon skipping if Drosha cleaves exon 5 from the pre-mRNA, thereby forcing splicing between exon 4 and exon 6. However, we found that an ∼eight-fold over-expression of Drosha resulted in significant 1.6-fold increase in exon 5 inclusion (Figure 4A and 4B).

Because Drosha cleaves *DGCR8* mRNA [40], over-expression of the protein can potentially lower Microprocessor activity rather than elevate it, which could explain the unexpected effect of Drosha in promoting exon 5 splicing. To address this possibility, we quantitated the abundance of *DGCR8* upon Drosha overexpression. We found no change in *DGCR8* abundance following Drosha overexpression (Figure 4D), presumably because Drosha abundance is not limiting DGCR8 cleavage. These results establish that a negative feed-back loop is not responsible for the observed change in exon 5 splicing and further support a potential role for Drosha in splicing.

To test more directly whether Drosha is involved in exon 5 alternative splicing by cleaving exon 5 or by simply binding to the exon, we uncoupled Drosha binding from cleavage by expressing TN Drosha in HEK-293T cells and assessed the effect of this cleavage-deficient form of Drosha on exon splicing by RT-PCR. TN Drosha expression, ∼five-fold higher than endogenous Drosha (Figure 4A and 4E) levels, caused a significant 2.4-fold increase in exon 5 inclusion relative to exon 5 skipped mRNA isoforms (Figure 4A and 4B). TN Drosha expression also affected Drosha miRNA biogenesis activity, as we observed a significant decrease in miR-126 RNA (Figure 3D) and an increase in *DGCR8* expression (Figure 4D), as expected [40], confirming that TN-Drosha was expressed at a functional level. This effect on splicing was specific to *eIF4H*, as TN Drosha did not affect the inclusion of another small alternative exon, exon 7 in *SMN2* (Figure S4), which is not predicted to form a hairpin. The fact that TN Drosha expression enhanced splicing to a greater degree than overexpression of wild-type Drosha suggests that sustained binding to the RNA, or binding without cleavage has a more robust effect on exon 5 splicing. We conclude that the Drosha-mediated enhancement of exon 5 splicing is most likely not mediated by cleavage of the pre-mRNA transcript, as overexpression of wild-type Drosha or TN Drosha resulted in similar improvements in splicing and neither resulted in alterations in total mRNA levels (Figures 3 and 4).

To further assess whether the increase in exon 5 splicing observed upon expression of TN Drosha was a result of Drosha binding, we depleted cellular Drosha by RNAi-mediated knockdown (Figure S5). We observed a significant increase in DGCR8 mRNA (Figure S5G), a substrate for Drosha cleavage, confirming a functional reduction in Drosha [40]. However, depletion of Drosha did not significantly affect the relative amount of exon 5 inclusion or total *eIF4H* mRNA (Figure S5E and S5F). Canonical miR-126 levels were only modestly reduced (Figure S5C and S5H), suggesting that miR-126 may be more stable relative to *DGCR8* mRNA. Together, our results suggest that Drosha can stimulate splicing, but is not required for the low level of basal exon 5 splicing. This result is consistent with a role for Drosha as a splicing enhancer.

We also considered the possibility that the increase in exon 5 splicing resulting from overexpression of TN Drosha and wild-type Drosha could be mediated by changes in miRNAs that are specifically targeted to sequences within exon 5. To test this idea, we manipulated miRNA biogenesis by overexpressing Dicer and TN Dicer in cells. We did not observe a change in *eIF4H* splicing when we expressed TN Dicer (Figure S6) or wild-type Dicer (Figure 4A, 4B and 4F), which suggests that *eIF4H* mRNA isoform abundance is not regulated by miRNAs (Figure S6 and 4). These results support a role for Drosha in stimulating exon 5 splicing in a manner that is distinct from its role in miRNA biogenesis.

We next performed immunoblot analysis of proteins isolated from HEK-293T cells transfected with or without TN Drosha and evaluated whether EIF4H protein abundance was altered in a Drosha-dependent manner, coincident with the change in exon 5 splicing. We found that the increase in *eIF4H* exon 5-containing mRNA isoforms that resulted from over-expression of TN Drosha also resulted in a significant increase (∼1.75 fold) in full-length EIF4H protein relative to the shorter isoform produced from mRNA lacking exon 5 (Figure 4G and 4H), The overall abundance of EIF4H protein was also reduced following TN Drosha expression (Figure 4I), perhaps reflecting differences in stability between the two isoforms or a decrease in translation of TN Drosha-bound mRNA.

### Drosha-induced splicing enhancement depends on exon 5 structure

**Figure 5:**
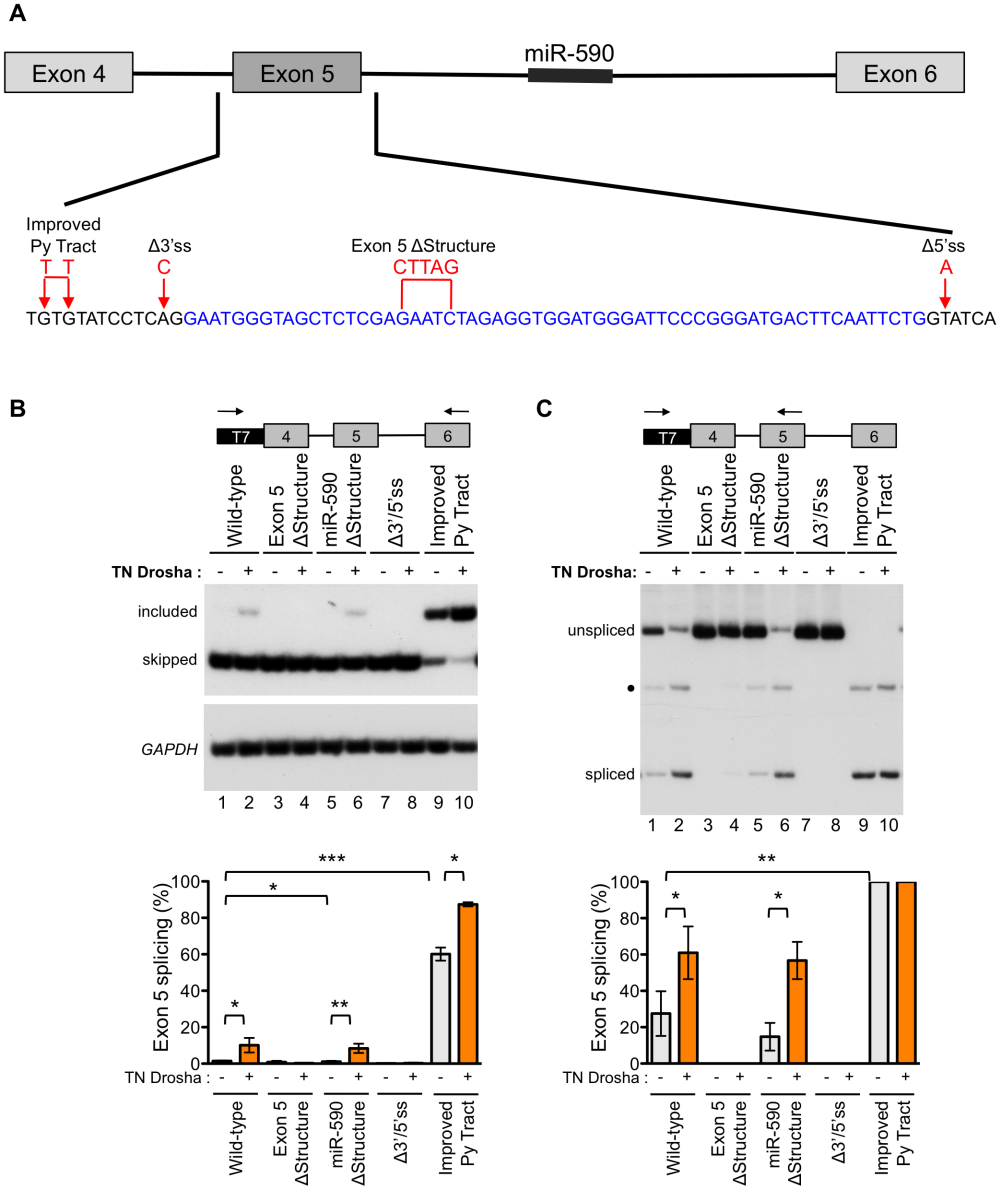
Exon 5 structure and splice sites affect the Drosha-mediated splicing enhancement. (A) A wild-type *eIF4H* minigene, containing exons 4–6 and the intervening introns, was mutated at the indicated positions to generate constructs with an improved pyrimidine (Py) tract, mutated 3′ and 5′ splice sites (Δ3′ss and 5′ss) or a disrupted secondary structure (ΔStructure). Blue, black and red letters designate exon 5, intronic sequences and mutations, respectively. (B) Radiolabelled RT-PCR analysis of minigene-derived *eIF4H* mRNA exons 4–6 (C) or exons 4–5 from HEK-293T cells transiently transfected with minigenes and with (+) or without TN Drosha (−). Primer locations are shown on the minigene maps above the gel images. Graphs show the percent of exon 5 splicing [included/(included+skipped)*100] or [spliced/(spliced+unspliced)*100]. * indicates statistical significance by Student's t-test. Values of p≤0.05 were considered significant. • indicates an uncharacterized band. Error bars represent SEM. For control-treated cells, n = 6. For TN Drosha treated cells, n = 3.

In order to better understand the role of Drosha in exon 5 splicing, we constructed a series of minigenes with mutations that alter the splicing or structure of the exon, and tested their effect on Drosha-induced splicing (Figure 5A). All mutations were designed to preserve the predicted hairpin structure of exon 5, except in the case of the exon 5 ΔStructure construct (Figure S7). Splicing of the minigene-derived RNA transcripts was analyzed by RT-PCR with primer pairs that specifically amplify minigene-derived RNA and that detect both the exon 5 included and skipped isoforms (Figure 5B) or detect the exon 5 included isoform and unspliced, intron 4 retained RNA (Figure 5C). In this way, we evaluated exon skipping, inclusion, and intron retention.

Fully-spliced mRNA that included exon 5 was not detectable from the wild-type *eIF4H* mini-gene transcripts, providing further evidence of the weak nature of exon 5 inclusion (Figure 5B, lane 1). However, splicing of exon 4 to exon 5 was observed (Figure 5C, lane 1), suggesting that inefficient splicing of exon 5 to exon 6 may limit exon 5 inclusion. The exon 5 structural mutation, which disrupts the hairpin, eliminated splicing of exon 4 to exon 5 (Figure 5C, lane 3). This result demonstrates that the structure of the exon is important for splicing and promotes, rather than inhibits the reaction (Figure 5). The *eIF4H* minigene with the mutation that improved the intron 4 polypyrimidine tract (Improved Py tract) produced predominantly exon 5 included mRNA and no intron retention (Figure 5B and 5C). Taken together, these results suggest that splicing of exon 5 to exon 6 limits exon 5 inclusion in the mRNA and that this limitation can be overcome by strengthening the 3′ splice site, which could define a potential role for Drosha in splicing enhancement via the hairpin.

To evaluate the role of Drosha in the control of exon 5 splicing in the context of the splicing and structural mutations, we co-transfected TN Drosha with the different minigenes. TN Drosha expression resulted in an seven-fold increase in exon 5 inclusion relative to skipping (Figure 5B, lanes 1, 2) and a 2.2-fold increase in splicing of exon 4 to exon 5 (Figure 5C, lanes 1, 2). The TN Drosha activity was dependent on intact splice sites and the hairpin structure (wild-type compared to Exon 5 ΔStructure, Δ3′/5′ss, Figure 5B and 5C), indicating that Drosha promotes exon 5 splicing in a structure-dependent manner. TN Drosha improved exon 5 inclusion of RNA from the improved Py tract-mutated minigene, but did not increase splicing of exon 4 to exon 5, as it was already completely spliced even in the absence of TN Drosha. These results suggest further that Drosha can act by improving splicing from exon 4 to exon 5 (Figure 5C, lanes 1, 2) and also may act to improve exon 5 inclusion by enhancing splicing of exon 5 to exon 6 (Figure 5B, lanes 9, 10). None of the mutations, with or without TN Drosha expression, affected the overall abundance of *eIF4H* mRNA (Figure S8), indicating that the observed changes in relative abundance of the mRNA isoforms were not due to significant changes in cleavage or stability of the RNA.

We also constructed a minigene with mutations that altered the structure of miR-590 in order to test the possibility that Drosha processing of, or binding to this downstream substrate could promote upstream splicing of exon 5, as has been reported for other intronic miRNAs [24]. We did not observe a change in exon 5 splicing when the miR-590 structure was disrupted with or without TN Drosha expression (Figure 5B and 5C). This result suggests that miR-590 does not affect Drosha-mediated enhancement of exon 5 splicing.

### Drosha binds preferentially to *eIF4H* exon 5

**Figure 6:**
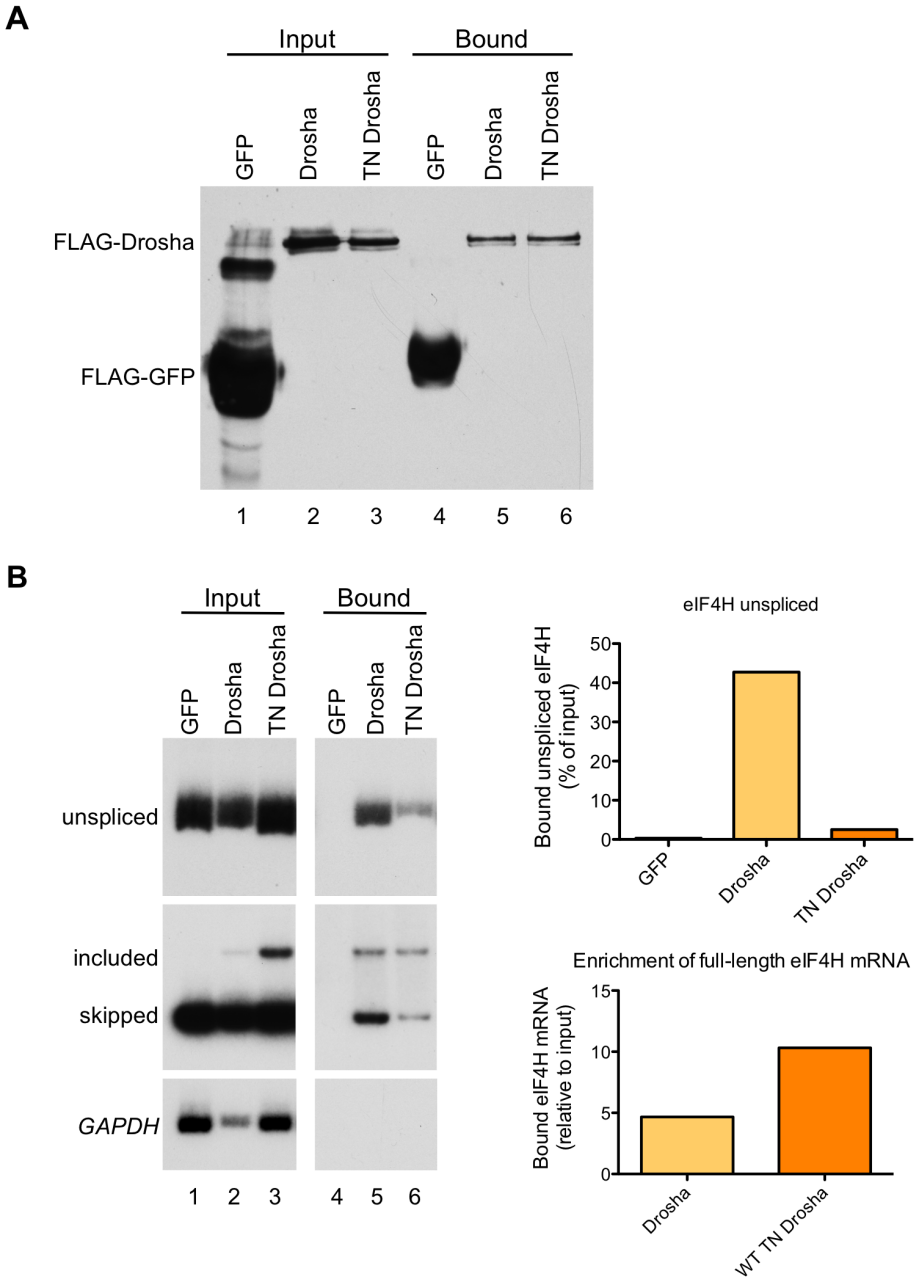
*eIF4H* exon 5 co-immunoprecipitates with Drosha and TN Drosha. (A) An immunoblot of the FLAG-tagged proteins present in the input (lanes 1–3) and bound to the anti-FLAG M2 magnetic beads (Sigma) (bound, lanes 4–6). (B) Radiolabelled RT-PCR analysis of *eIF4H* unspliced RNA and mRNA in the input fraction (lanes 1–3) and bound to FLAG-tagged proteins (lanes 4–6) from HEK-293T cell lysates that were transiently transfected with *eIF4H* WT minigene and FLAG-tagged GFP (control), Drosha, or TN Drosha. *GAPDH* is a negative control. The top graph (*eIF4H* unspliced) shows the percent of unspliced RNA that was bound to the FLAG-tagged proteins, corrected for dilutions of Input relative to bound samples [bound/(input * dilution correction factor)]. The bottom graph shows the enrichment of exon 5 splicing in the bound fraction relative to input [(included/skipped)_bound_/(included/skipped* dilution correction factor)_input_]. Drosha values are an average of two independent experiments.

To test whether the effect of Drosha on exon 5 splicing is due to a direct interaction between the protein and the exon, we performed co-immunoprecipitation experiments with Drosha and TN-Drosha. For this, we over-expressed FLAG-tagged Drosha or TN Drosha with the *eIF4H* wild-type minigene in HEK-293T cells, immunoprecipitated the proteins from the cellular lysates (Figure 6A), and assayed for co-immunoprecipitation of exon 5 unspliced mRNA and both mRNA isoforms with Drosha (Figure 6B). Unspliced *eIF4H* pre-mRNA and exon 5 included and skipped isoforms were bound by Drosha and TN Drosha. However, there was a dramatic 4.5–10-fold enrichment of exon 5 included mRNA relative to skipped isoforms in the bound fraction relative to the input fraction (Figure 6B), an effect that was enhanced in the TN Drosha immunoprecipitates. TN Drosha bound to *eIF4H* unspliced pre-mRNA to a lesser degree than wild-type Drosha, possibly because TN Drosha enhances exon 5 splicing more than Drosha (Figure 4A and 4B) and thus, less unspliced, pre-mRNA is expected to be present in the samples. These results indicate a preferential binding of Drosha to *eIF4H* exon 5 supporting a role for Drosha in splicing that involves direct binding to the exon.

### Drosha has a direct effect on *eIF4H* splicing

**Figure 7:**
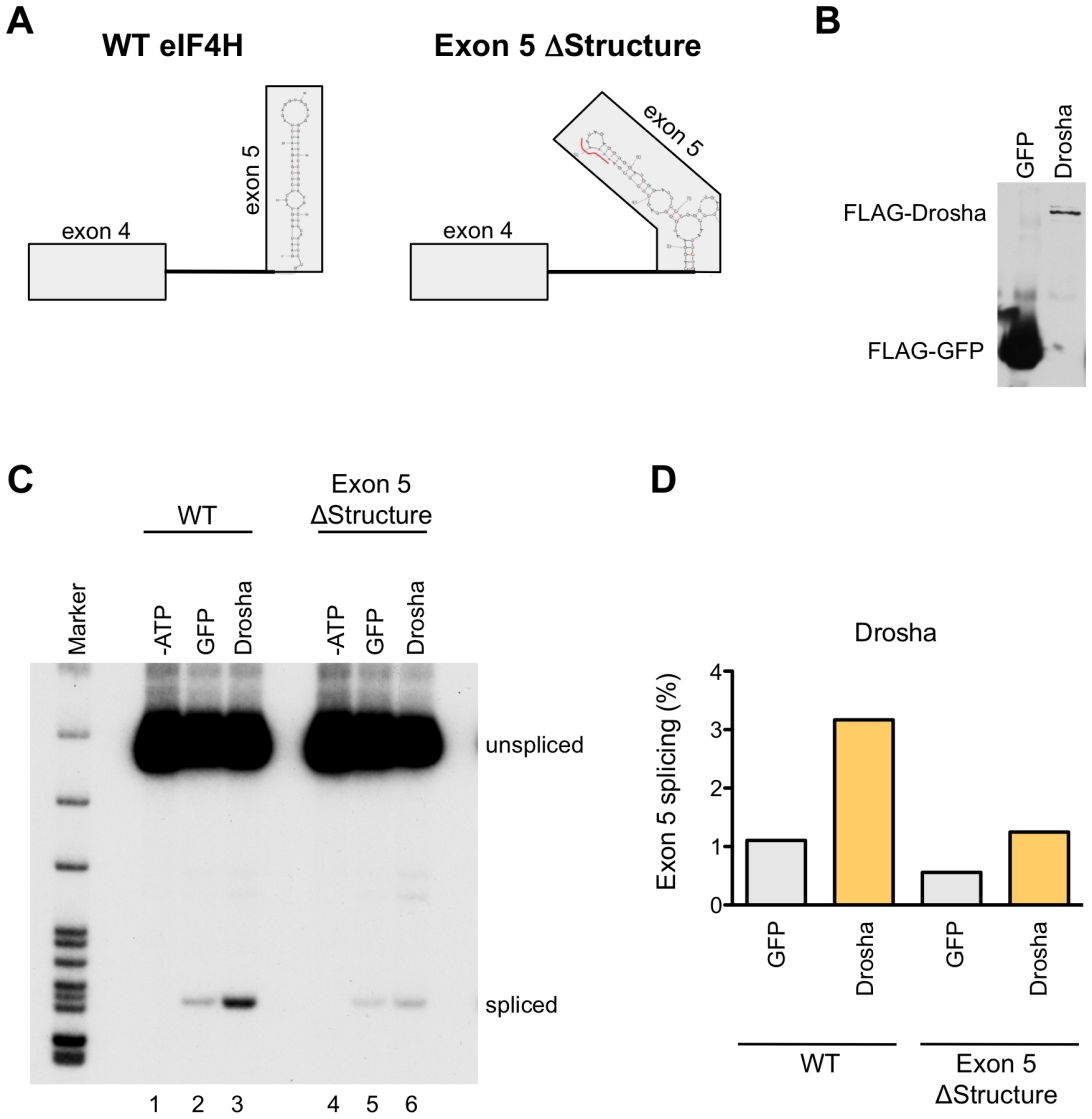
Drosha enhances exon 5 splicing directly. (A) The wild-type (WT) or Exon 5 ΔStructure minigenes templates that were used to generate RNA for the *in vitro* splicing assay are shown with the predicted exon 5 structures. Boxes represent exons and lines are introns. The red line in the ΔStructure mutant indicates the nucleotides that were mutated. (B) Immunoblot of FLAG-tagged GFP and Drosha that bound to anti-FLAG M2 magnetic beads (Sigma) and were used in the *in vitro* splicing assays. (C) Radiolabelled RT-PCR analysis of the RNA produced from *in vitro* splicing reactions of WT and Exon 5 ΔStructure RNA. RNA species (spliced and unspliced) are indicated. Splicing reactions were carried out in HeLa nuclear extracts in the absence of ATP (-ATP, lanes 1 and 4), or in the presence of ATP and supplemented with FLAG-tagged GFP (lanes 2 and 5), or Drosha (lanes 3 and 6). (D) Graph shows the percent of exon 5 splicing [spliced/(spliced+unspliced)*100].

To further confirm the role of Drosha in *eIF4H* splicing, we developed a cell-free assay for *eIF4H* exon 5 splicing that uncouples splicing from other cellular processes that could alter splicing, thereby allowing us to test whether Drosha has a direct role in *eIF4H* exon 5 splicing (Figure 7). Because intron 5 is too long to efficiently transcribe *in vitro*, we used an RNA splicing substrate comprised of exons 4 and exon 5 and the intervening intron 4 from wild-type and exon 5 ΔStructure *eIF4H* minigenes (Figure 7A). We *in vitro* transcribed the RNA and assessed splicing of this substrate in HeLa nuclear extract under splicing conditions in the presence or absence of immunoprecipitated FLAG-Drosha (Figure 7C and 7D) and FLAG-TN Drosha (Figure S9). Incubation of the RNA with HeLa nuclear extracts resulted in an ATP-dependent product of the expected size and sequence of correct exon 4 to exon 5 splicing. The addition of Drosha or TN Drosha to the reactions resulted in a dramatic enhancement of exon 5 splicing relative to nuclear extract alone or with FLAG-GFP (Figures 7C and S9). Disrupting the hairpin structure of exon 5 resulted in a 2–2.5-fold reduction in exon 5 splicing (Figures 7D and S9B), and lowered the effect of Drosha on splicing, as expected if Drosha mediates splicing enhancement through the hairpin structure. Our results demonstrate that Drosha and TN Drosha promote *eIF4H* splicing via the hairpin structure.

## Discussion

Here, we report on the alternatively spliced *eIF4H* exon 5, which has a predicted hairpin structure that resembles a Drosha cleavage substrate. We show that Drosha not only can bind and cleave this exon, but it also can enhance splicing of exon 5. We do not detect appreciable amounts of cleavage of the exon by Drosha in cultured cells under normal conditions (Figures 3 and S3). Rather, we find that the primary effect of Drosha binding to the exon is the stimulation of exon 5 splicing, which occurs in a cleavage-independent but structure-dependent manner both in cell-free (Figure 7) and cell-based assays (Figures 4, 5 and 6)

Taken together, our results identify an RNA sequence that has the potential to function as an exon or a miRNA, and demonstrate that Drosha can mediate both of these functions. This finding suggests that Drosha could be a key factor in the coordination and co-regulation of intragenic miRNA biogenesis and splicing. *eIF4H* exon 5 splicing may exemplify a more wide-spread mode of alternative splicing regulation that is controlled by Drosha. The unusual multi-functional exon 5 sequence may also be an indication that the association of the spliceosome and Drosha has resulted in the creation of an exon as a result of recruitment of the spliceosome by Drosha or vice versa, the creation of a miRNA by recruitment of Drosha by the spliceosome.

One possibility is that Drosha has different functions depending on its binding partners and the context of its binding. Differential activity of Drosha has been suggested by its poor cleavage activity when it has been co-purified with a large protein complex that includes a number of specific splicing factors [2]. This alternative Drosha complex may have a different function in the cell than when Drosha is part of the Microprocessor complex.

Numerous studies have reported interactions between the spliceosome and the Microprocessor components [2], [19], [20], which suggests an intimate association between the miRNA cleavage and splicing of the pre-mRNA that is carried out by these complexes. Recruitment of the spliceosome is required for intron removal and exon ligation. However, the presence of the Microprocessor during the splicing reaction, by virtue of the Microprocessor's association with the spliceosome, could lead to cleavage of mRNA hairpins near splice sites, or alter splicing patterns. Indeed, widespread cleavage by the Microprocessor has been reported, and does not necessarily result in the production of miRNAs, suggesting that Drosha can cleave RNA hairpins in a manner that is more indiscriminate than previously appreciated [30], [31], [41], [42]. Given that RNA hairpins are the most common RNA structure [43], cleavage by the Microprocessor has the potential to have a significant impact on mRNA expression, especially given its interaction with the spliceosome. Mechanisms to control widespread cleavage of RNA by the Microprocessor must be in place to ensure mRNA expression. Our demonstration that Drosha can function as a splicing factor may suggest that modulation of Drosha activity regulates and coordinates cleavage and splicing.

Widespread and frequent cleavage of pre-mRNA transcripts by the Microprocessor is likely inhibited by other mechanisms dictating pre-mRNA processing to avoid massive down-regulation of gene expression. For example, the expression levels of the Microprocessor may determine, in part, the efficiency and specificity of Microprocessor substrate cleavage and prevent off-target cleavage [44]. Canonical splicing factors may also contribute to the regulation of Microprocessor-associated cleavage of intragenic RNA hairpins. Perhaps, when Drosha is associated with the spliceosome, it functions in a cleavage-independent manner. Additionally, splice site strength could dictate the processing events. For example, when splicing is efficient, the Microprocessor may not have the opportunity to cleave nearby hairpins due to splicing efficiency. In contrast, if splicing does not occur efficiently at a particular splice site, the Microprocessor may cleave nearby hairpins, possibly acting as a proofreading mechanism for inefficient splicing events. This system may have evolved to select against or regulate splicing or mis-splicing events that are characterized by weak splice sites. In the case of *eIF4H* exon 5, the association of the spliceosome with the Microprocessor may have led to the activation of an alternative exon or the creation of low abundance, miRNAs.

The collaboration between the spliceosome and Microprocessor is evident in our result that exon 5 splicing is enhanced *in vitro* and in cells by the overexpression of Drosha and by expression of the catalytically inactive TN Drosha (Figures 4, 7 and S9). The influence of splicing and Microprocessor activity on each other is also apparent when splicing efficiency is altered. Improvement of the polypyrimidine tract resulted in a dramatic increase in exon 5 splicing (Figure 5). This improvement in a core splice site signal may eliminate the dependence of splicing on Microprocessor recruitment via the hairpin, though Drosha can still further enhance splicing (Figure 5B).

A role for Drosha in splicing is not currently recognized as a common phenomenon, perhaps because the effect may be masked by competitive miRNA processing which could result in cleavage of the spliced isoforms that are enhanced by Microprocessor binding. This potential competition may also explain why knockdown of Drosha did not alter *eIF4H* exon 5 splicing, as there would be a reduction in mRNA cleavage by Drosha into the canonical 5p and 3p/pre-miRs that may preserve the exon 5 included mRNA isoforms. Although we cannot rule out a more robust competition between Microprocessor cleavage of exon 5 and splicing of exon 5 under certain circumstances, we do not find evidence for such competition in the cell system that we tested here. We find that Drosha can cleave exon 5 (Figure 2), but this does not appear to be a common event, as the corresponding small RNAs are not detectable by Northern blot (Figure S3) and we did not observe a change in mRNA abundance in the presence of TN Drosha (Figure 3). Previous studies have suggested a role for the Microprocessor in splicing. One report found that DGCR8 binds to many different mRNAs that have hairpin structures including alternatively spliced exonic sequences [31], [41]. Although analysis of alternative splicing changes in mouse cells lacking DGCR8 revealed many instances where exon inclusion is up-regulated, consistent with competition between splicing and miRNA processing, there were also numerous alternative exon isoforms that are down-regulated, which could be indicative of the enhancement of splicing by the Microprocessor which is absent in the DGCR8 knock-out cells [31]. Other groups have also observed a decrease in specific mRNAs following DGCR8 knockout [42]. These findings further suggest that the Microprocessor has activity as a splicing enhancer.

Regulation of exon 5 splicing may be physiologically relevant, as EIF4H plays a critical regulatory role in eIF4A helicase activity during translation initiation [45]–[47]. An increase in expression of the *eIF4H* mRNA isoform that includes exon 5 is associated with gastrointestinal cancer and has been implicated in cell proliferation and carcinogenesis [46], [47], indicating that control of exon 5 splicing is critical for proper cellular function. Indeed, according to the online cancer database Oncomine version 4.4.4 (Compendia Bioscience Inc., Ann Arbor, MI, USA), Drosha is overexpressed in a number of cancers including gastrointestinal tumors. Thus, Drosha may correlate with and potentially contribute to the upregulation of the exon 5 included isoform of *eIF4H* that has been observed in gastrointestinal cancers. Furthermore, if Drosha regulation of alternative splicing is a more wide-spread function of the protein, it is possible that changes in Drosha expression or activity could affect broad alternative splicing programs. Drosha levels have been reported to vary in different cell types [48] and disease conditions [49], which could result in splicing changes in addition to changes in miRNA and mRNA abundance that result from Drosha cleavage.

Overall, our results reveal a role for Drosha in splicing of an alternative exon that forms a pre-miRNA-like hairpin structure. Given the prevalence of RNA hairpins in pre-mRNA [43], the phenomenon of co-regulation of splicing and cleavage by the Microprocessor could be a prevalent mechanism for controlling gene expression, as exemplified in the case of *eIF4H*, described here.

## Materials and Methods

### Sequence analysis

Sequences alignments were generated using the MultAlin algorithm [50] with sequences from *Homo sapiens* (GRCh37), *Mus musculus* (GRCm38), *Monodelphis domestica* (BROADO5), and *Gallus gallus* (Galgal4). RNA structures were predicted using mfold RNA folding algorithm [51].

### Plasmids

pCK-FLAG-TN Drosha and pCK-FLAG-TN Dicer were generous gifts from V. Narry Kim. pCK-FLAG-Drosha and pCK-FLAG-Dicer were previously generated in our lab [37]. FLAG-DGCR8 (Addgene) was purchased. The wild-type *eIF4H* minigene, comprised of exons 4–6 and the intervening introns, was generated by amplifying human genomic DNA with LongAmpTaq DNA polymerase (New England Biolabs). The amplicon was inserted into the pTarget vector (Promega) as per manufacturer's instructions. Mutations were introduced with the QuikChange Lightning mutagenesis kit (Agilent). Primer sequences are provided in Table S1. All plasmid constructs were sequenced to verify the correct insertion sequence.

### *In vitro* transcription, miRNA processing and splicing

T7 DNA templates were generated from human genomic DNA or *eIF4H* minigenes by PCR with GoTaq Green Master Mix (Promega). Primer sequences are provided in Table S1. For the *eIF4H* substrates used in the *in vitro* miRNA processing experiments, the last 101 nts of intron 4 through the first 100 nts of intron 5 were included in the transcript. For the *eIF4H* transcripts used in *in vitro* splicing assays, the transcript contained exons 4 and 5 and the intervening intron. For RNA substrates used in miRNA processing, 150 nts to 100 nts of the intronic sequences flanking the exon or pre-miRNA were included in the transcript (see Table S1 for primer sequences). All RNA substrates used in *in vitro* miRNA processing were *in vitro* transcribed using T7 RNA polymerase (Promega), transcription buffer (Promega), with 10 mM DTT, 0.5 mM A, C, and G, 0.02 mM U, α-^32^P UTP and RNase inhibitor (Promega), whereas *in vitro* transcription reactions with transcripts used in *in vitro* splicing had 0.5 mM G and 0.5 µM 7Me-GpppG cap analog (New England Biolabs). The reactions were incubated for 1 hr at 37°C then treated with RQ1 DNase (Promega) for 30 min at 37°C. Reaction products were separated on 5% denaturing PAGE gels, and then excised and eluted from the gel in 0.3 M sodium acetate and 0.1% SDS (w/v), followed by phenol extraction, ethanol precipitation and were finally reconstituted in water.

For *in vitro* miRNA processing, FLAG-tagged GFP, DGCR8, Drosha and Dicer or dominant negative (TN) versions of Drosha and Dicer were transfected into HEK-293T cells, as previously described [37], and the FLAG-tagged proteins were isolated on M2-FLAG beads (Sigma). *In vitro* processing was carried out as previously described [37] except that 12 µg of each FLAG-tagged plasmid was transiently transfected into the cells and 10 fmol of *in vitro* transcribed RNA was used per reaction. Reactions were incubated for 2 hr at 30°C, and the products were separated on 8.4 M urea, 12% denaturing PAGE gels.

For *in vitro* splicing assays, HEK-293T cells were transfected with FLAG-tagged GFP, Drosha or TN Drosha and the proteins were collected in the same manner as for *in vitro* processing as previously described [37]. FLAG-Drosha, TN Drosha or GFP bound to anti-FLAG magnetic beads (Sigma) in lysis buffer [37], or lysis buffer alone, were combined with 10 fmol of *in vitro* transcribed RNA and HeLa nuclear extract under splicing conditions (32 mM HEPES, 3 mM MgCl_2_, 2.6% polyvinyl alcohol, 1X buffer D (20 mm HEPES-KOH, pH 8; 100 mm KCl; 0.2 mm EDTA; 20% (v/v) glycerol), 73 mM KCl) with or without 0.5 mM ATP and 4 mM creatine phosphate. The reactions were incubated for 2 hrs at 30°C. Reactions were stopped with stop buffer (0.3 M sodium acetate and 0.1% (w/v) SDS) and the RNA was phenol extracted followed by ethanol precipitation. All RNA was reverse transcribed with Goscript RT (Promega) using a reverse primer to exon 5 using. PCR with GoTaq Green master mix (Promega) was performed with a forward primer specific to the pTarget plasmid sequence that was present in the *in vitro* transcribed RNA and the reverse primer to exon 5. Spliced products were confirmed by sequencing.

### 5′/3′ RACE and sequencing

The *in vitro* processed RNA products in the range of 30–80 nts were excised from the gel, eluted, phenol/chloroform extracted, ethanol precipitated and resuspended in water. A 3′ linker, Linker 1 (Integrated DNA Technology), was added either to the excised *in vitro* processed products or total RNA from HEK-293T cells using RNA ligase 2 truncated (New England Biolabs) as per the manufacturer's instructions. A 5′ linker, M.R.S. linker (Integrated DNA Technology), was ligated to the 3′-linkered-RNA, using RNA ligase 2 (New England Biolabs) as per the manufacturer's instructions. The linkered RNA was reverse transcribed with a primer to the 3′ linker using GoScript Reverse Transcriptase (Promega). 5′ ends were identified using a reverse primer to *eIF4H* exon 5 and a forward primer to the 5′ linker. 3′ ends were determined using a forward primer to *eIF4H* exon 5 and a reverse primer to the 3′ linker. Primer sequences are provided in Table S1. PCR products were separated on 12% native PAGE gels, extracted, eluted, ethanol precipitated, resuspended and ligated into the pGEMEasy-T Vector (Promega) and sequenced at Northwestern University's Genomics core.

### Cell culture and transfections

HEK-293T and HeLa cells were cultured in Dulbecco's modified Eagle's medium supplemented with 10% fetal bovine serum. Plasmids were transfected with Lipofectamine 2000 (Invitrogen) as per the manufacturer's instructions. For all experiments involving expression plasmids, RNA was harvested 48 hrs after transfection. Drosha RNAi experiments were performed as previously described [31]. RNA was collected using Trizol (Invitrogen) per the manufacturer's instructions.

### Northern blot analysis

Total RNA was collected with Trizol (Invitrogen) from untreated HeLa cells or HeLa cells transiently transfected with wild-type *eIF4H* minigene using Lipofectamine 2000 (Invitrogen) as per the manufacturer's instructions. RNA (30 µg) was separated on an 8% denaturing PAGE gel and then transferred by electoblotting to Bright-Star-Plus membrane (Ambion), cross-linked and then blocked in Ultra-Hyb buffer (Ambion) as per the manufacturer's instructions. RNA was detected using a custom-designed miRCURY LNA detection probe (Exiqon) targeting the first 18 nts of *eIF4H* exon 5, that was end labeled with ^32^P-γ-ATP and incubated overnight. The membrane was washed twice with low stringency wash (2× SSC, 0.1% SDS) followed by two washes with high stringency wash (0.1XSSC, 0.1% SDS). Membranes loaded with the same samples were probed with a ^32^P-γ-ATP end-labeled miR-590 miRCURY LNA detection probe (Exiqon 38686-00).

### Immunoprecipitation

Immunoprecipitation of FLAG-tagged proteins and analysis of co-precipitated RNAs derived from HEK-293T cells transiently transfected with FLAG-Tagged GFP, Drosha, or TN Drosha and wild-type *eIF4H* minigene was performed as previously described [37].

### RT-PCR

Reverse transcription was performed using GoScript Reverse Transcriptase (Promega) as per manufacturer's instructions. The same kit was used to reverse transcribe small RNAs with gene-specific stem-loop primers as previously described [37] or linker-specific primers. PCR was performed with GoTaq Green Master Mix (Promega) and α-^32^P-dCTP. Various PCR cycle numbers were tested to insure amplification in the linear range. Amplification products from stem-loop PCR of small RNAs were separated on 12% native PAGE gels and mRNA products on 6% native PAGE gels. Products were quantitated using a Typhoon 9400 Variable Mode Imager (GE Healthcare), with the exceptions of 3p/pre-miR following expression of TN Drosha and the unspliced pre-mRNA levels of *eIF4H* in the immunoprecipitation assays, which were quantitated using NIH Image J software. SMN PCR reactions were incubated with DdeI (New England Biolabs). for 1 hr at 37°C and subsequently resolved on 6% Native PAGE gels. DdeI specifically cleaves *SMN2* but not *SMN1* allowing for separation of *SMN1* and *SMN2*.

### Quantitative real-time PCR

Total RNA was collected from HEK-293T cells that were transiently transfected with pCK-FLAG-TN Drosha using Lipofectamine 2000 (Invitrogen) and mock-treated control cells. RNA was reverse transcribed with GoScript Reverse Transcriptase (Promega). PCR was performed with Taqman gene expression master mix (Applied Biosystems) with probes to *eIF4H* (Hs01586164_g1 Applied Biosystems) or Human *ACTB* (4333762F Applied Biosystems) on an Applied Biosystems (ABI) 7500 Real-Time PCR System using the ABI 7500 detection software. Results were analyzed with the ΔΔCt method [52].

### Immunoblot analysis

Protein was collected from cells transiently transfected with Lipofectamine 2000 (Invitrogen) alone, FLAG-Tagged GFP, pcK-FLAG-Drosha or pCK-FLAG-TN Drosha in 2X Laemmli sample buffer. Proteins were separated on a 5% stacking, 12% resolving SDS-PAGE gels and transferred to FL-Immobilon membrane (Millipore) and probed with rabbit-anti-eIF4H (Cell Signaling) or Mouse-Anti-FLAG (Sigma). β-Actin was detected with mouse-anti-β-actin (Sigma). Proteins were visualized with Luminata Classico western HRP substrate (Millipore). Quantitation was preformed using NIH ImageJ software.

### Statistics

All statistics were performed using unpaired or paired Student's t-test. Results were considered significant when p≤0.05.

## Supporting Information

Figure S1 Predicted structures of *eIF4H*, *SMN1* and *APP* alternative exons and miR-590. The predicted RNA secondary structures of three alternative human exons *eIF4H* exon 5, *SMN1* exon 7, and *APP* exon 8 and the secondary structure of the canonical miRNA miR-590, as determined by the RNA folding software mFold [51]. The free energies are provided in kcal/mol. All structures predicted by mfold are shown, with the exception of *APP* in which a second, highly similar structure was also predicted (not shown).
(TIF)

Figure S2 The Microprocessor excises *eIF4H* exon 5. (A) The products of 3′ and 5′ RACE analysis of RNA from *in vitro* processing reactions and whole HEK-293T cells are shown. Red letters indicate introns. Black letters indicate exon 5. Gray arrows show the locations of gene specific primers for the RACE assays. Dashes represent sequences that were not detected in those clones. ✓ marks indicate the source of the RACE products. (B) Arrow heads indicate 5′ cleavage sites (black) and 3′ cleavage sites (green) on the exon 5 structure. The structure consists of exon 5 and flanking intronic sequences. The 5′ and 3′ splice sites (ss) are indicated by black arrows.
(TIF)

Figure S3 A putative exon 5-derived pre-miRNA is detectable in HeLa cells. Northern blot of endogenous RNA from HeLa cells or HeLa cells transfected with an *eIF4H* minigene (structure shown in top panel) probed with an LNA probe to exon 5 (left panel), which shows the presence of an excised exon 5 species (pre-miR). Molecular weight markers are shown on the left. A 60 nt size marker consisting of the exon 5 sequence is shown, indicating the size of the exon and the functionality of the LNA probe. Another membrane containing the same samples was probed with an LNA probe to miR-590-5p (right panel). Both pre- and mature miR-590-5p are detected.
(TIF)

Figure S4 TN Drosha does not affect *SMN2* exon 7 splicing. Radiolabelled RT-PCR analysis of endogenous *SMN2* exon 7 splicing in control HEK-293T cells or cells that were transiently transfected with TN Drosha. *SMN1* and *SMN2* were amplified via RT-PCR and digested with DdeI, which only digests *SMN2*. Graph shows the percent of *SMN2* exon 7 splicing [+Exon 7/(+Exon 7+ΔExon 7)*100], n = 3. Error bars represent SEM.
(TIF)

Figure S5 Drosha knockdown does not affect *eIF4H* exon 5 splicing. (A) An immunoblot of Drosha expression in HeLa cells treated with control siRNA (siControl) or Drosha siRNA (siDrosha). β-actin is a loading control. (B) Radiolabelled RT-PCR analysis of *eIF4H* exon 5 splicing and expression of *Drosha, DGCR8* and *GAPDH*, a loading control following Drosha RNAi. (C) Radiolabelled stem-loop RT-PCR analysis of a canonical miRNA, miR-126, expression, sno65 is a loading control. Graphs show quantitation of (D) Drosha mRNA levels relative to *GAPDH* (*Drosha/GAPDH*), (E) ratio of exon 5 splicing (included/skipped), (F) *eIF4H* abundance relative to *GAPDH* [(included+skipped)/*GAPDH*], (G) *DGCR8* expression, a non-canonical target of the Microprocessor, relative to *GAPDH (DGCR8/GAPDH)* and (H) miR-126 expression relative to sno65 (mir-126/sno65). * indicates statistical significance by Student's t-test where p≤0.05, n = 3. Bars represent SEM.
(TIF)

Figure S6 TN Dicer does not affect splicing of *eIF4H* exon 5. (A) Radiolabelled RT-PCR analysis of *eIF4H* splicing in control or TN Dicer treated HEK-293T cells. Graph shows percent of exon 5 splicing [included/(included+skipped)*100]. (B) Stem-loop RT-PCR analysis of miR-16 expression in control and TN Dicer treated cells. Graph shows miR-16 expression relative to sno65 (miR-16/sno65). miR-16 is a positive control and sno65 is a loading control. * indicates statistical significance by Student's t-test where p≤0.05, n = 6. Bars represent SEM.
(TIF)

Figure S7 Predicted structures of exon 5 and miR-590 RNA from mutated minigenes. (A) The hairpin structure of *eIF4H* exon 5 was preserved in all mutated forms of the minigene with the exception of the mutations that intentionally altered the structure of exon 5 (exon 5 ΔStructure) or (B) miR-590 (miR-590 ΔStructure). Each minigene is shown with the exon and flanking intronic sequences. Red arrows and lines indicate the specific mutation for individual minigenes.
(TIF)

Figure S8 Minigene mutations do not alter *eIF4H* abundance. The graph indicates overall *eIF4H* abundance relative to *GAPDH* in HEK-293T cells transiently transfected with the indicated minigenes and with or without TN Drosha [(included+skipped)/*GAPDH*]. For minigenes alone, n = 6, for minigenes co-transfected with TN Drosha n = 3.
(TIF)

Figure S9 TN Drosha enhances exon 5 splicing in vitro. (A) Immunoblot of FLAG-tagged GFP and TN Drosha probed with anti-FLAG antibody. (B) Radiolabelled RT-PCR analysis of RNA from *in vitro* splicing reactions of WT and Exon 5 ΔStructure RNA. Splicing reactions were carried out in HeLa nuclear extracts (-, lanes 1 and 4), or nuclear extracts supplemented with FLAG-tagged GFP (lanes 2 and 5), or TN Drosha (lanes 3 and 6). Unspliced and spliced (exon 4 to 5) RNA is indicated. Graph shows the percent of exon 5 splicing [spliced/(spliced+unspliced)*100].
(TIF)

Table S1 Primer sequences. Primers used in this study are shown 5′ to 3′.
(PDF)
