# Supplementary figures and images for: Drosha Promotes Splicing of a Pre-microRNA-like Alternative Exon
Source: PLoS Genet. 2014 May 1;10(5):e1004312. doi: 10.1371/journal.pgen.1004312 (PMC4006729; doi:10.1371/journal.pgen.1004312)

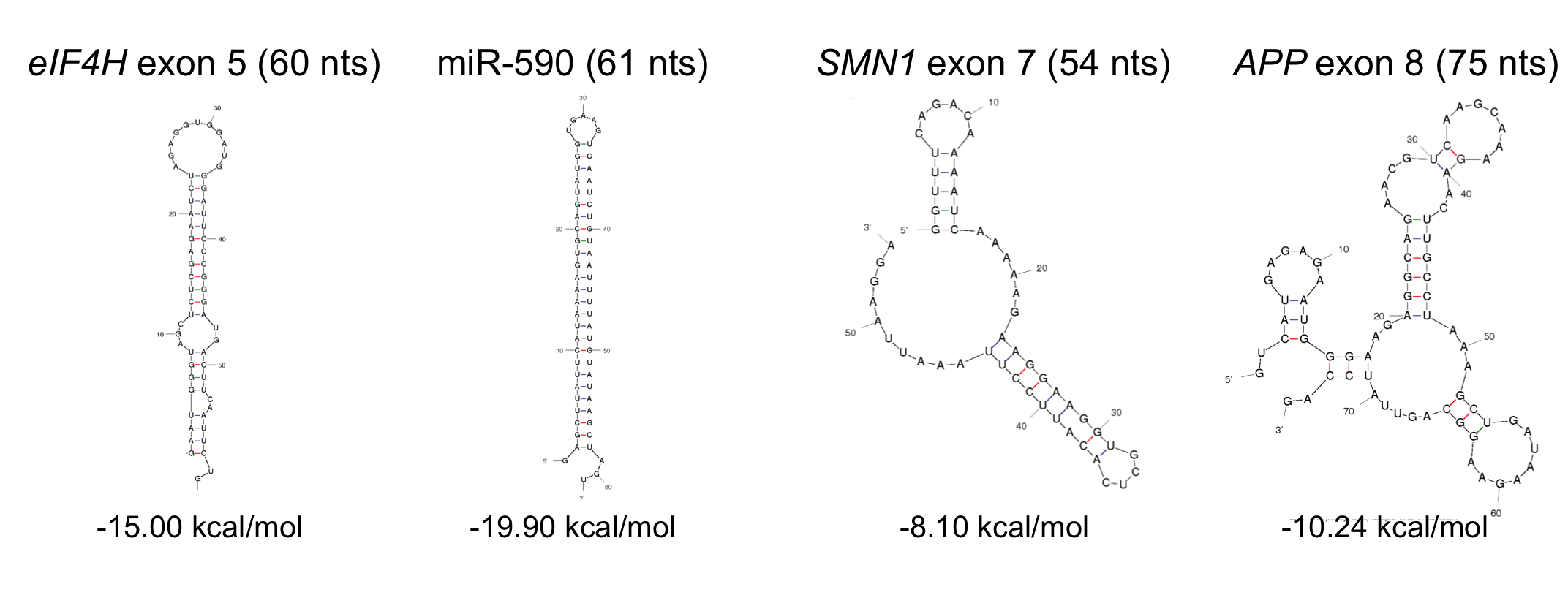

Supplement: Figure S1 — Predicted structures of eIF4H, SMN1 and APP alternative exons and miR-590. The predicted RNA secondary structures of three alternative human exons eIF4H exon 5, SMN1 exon 7, and APP exon 8 and the secondary structure of the canonical miRNA miR-590, as determined by the RNA folding software mFold [51]. The free energies are provided in kcal/mol. All structures predicted by mfold are shown, with the exception of APP in which a second, highly similar structure was also predicted (not shown). (TIF) [file pgen.1004312.s001.tif]

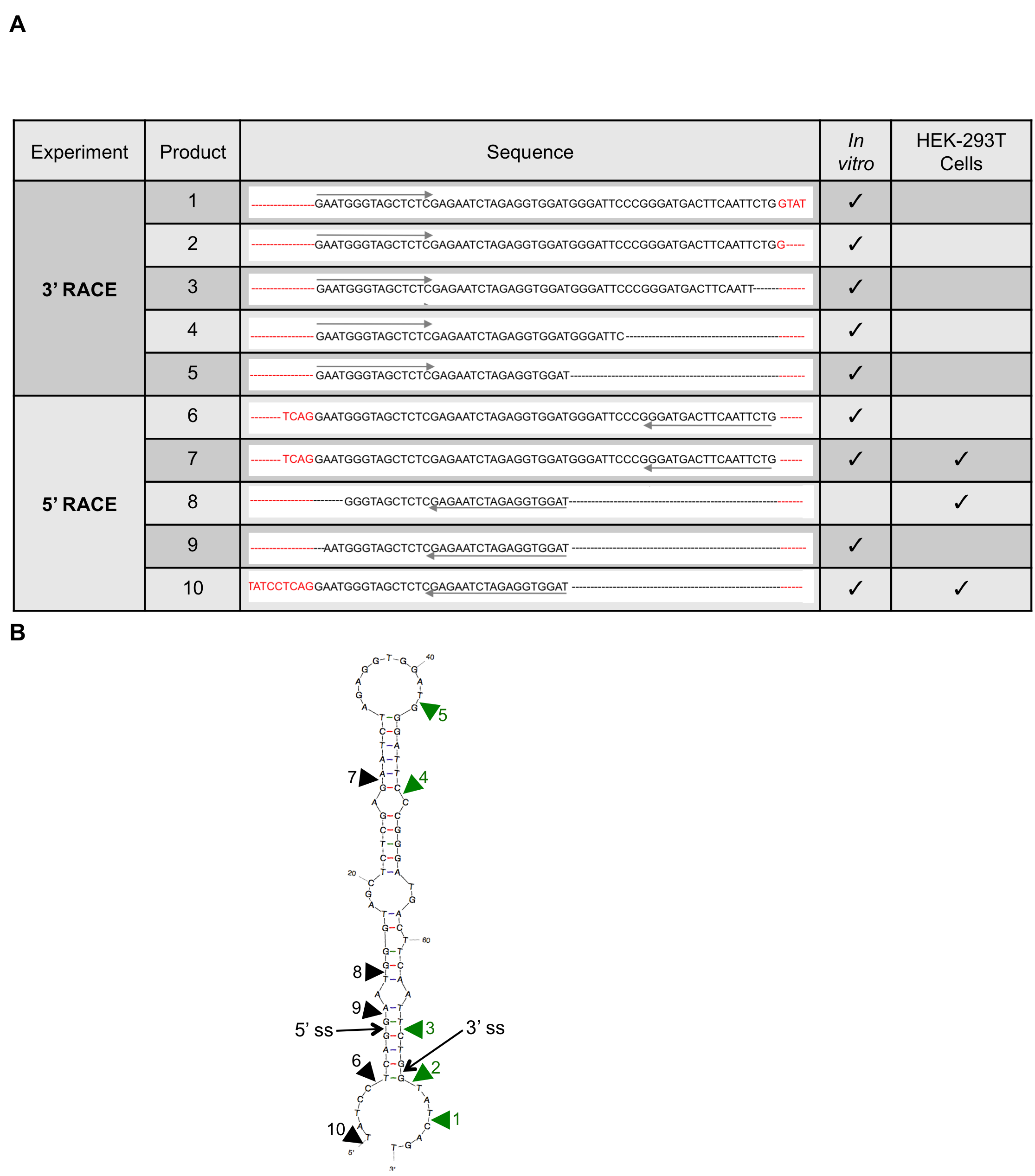

Supplement: Figure S2 — The Microprocessor excises eIF4H exon 5. (A) The products of 3′ and 5′ RACE analysis of RNA from in vitro processing reactions and whole HEK-293T cells are shown. Red letters indicate introns. Black letters indicate exon 5. Gray arrows show the locations of gene specific primers for the RACE assays. Dashes represent sequences that were not detected in those clones. ✓ marks indicate the source of the RACE products. (B) Arrow heads indicate 5′ cleavage sites (black) and 3′ cleavage sites (green) on the exon 5 structure. The structure consists of exon 5 and flanking intronic sequences. The 5′ and 3′ splice sites (ss) are indicated by black arrows. (TIF) [file pgen.1004312.s002.tif]

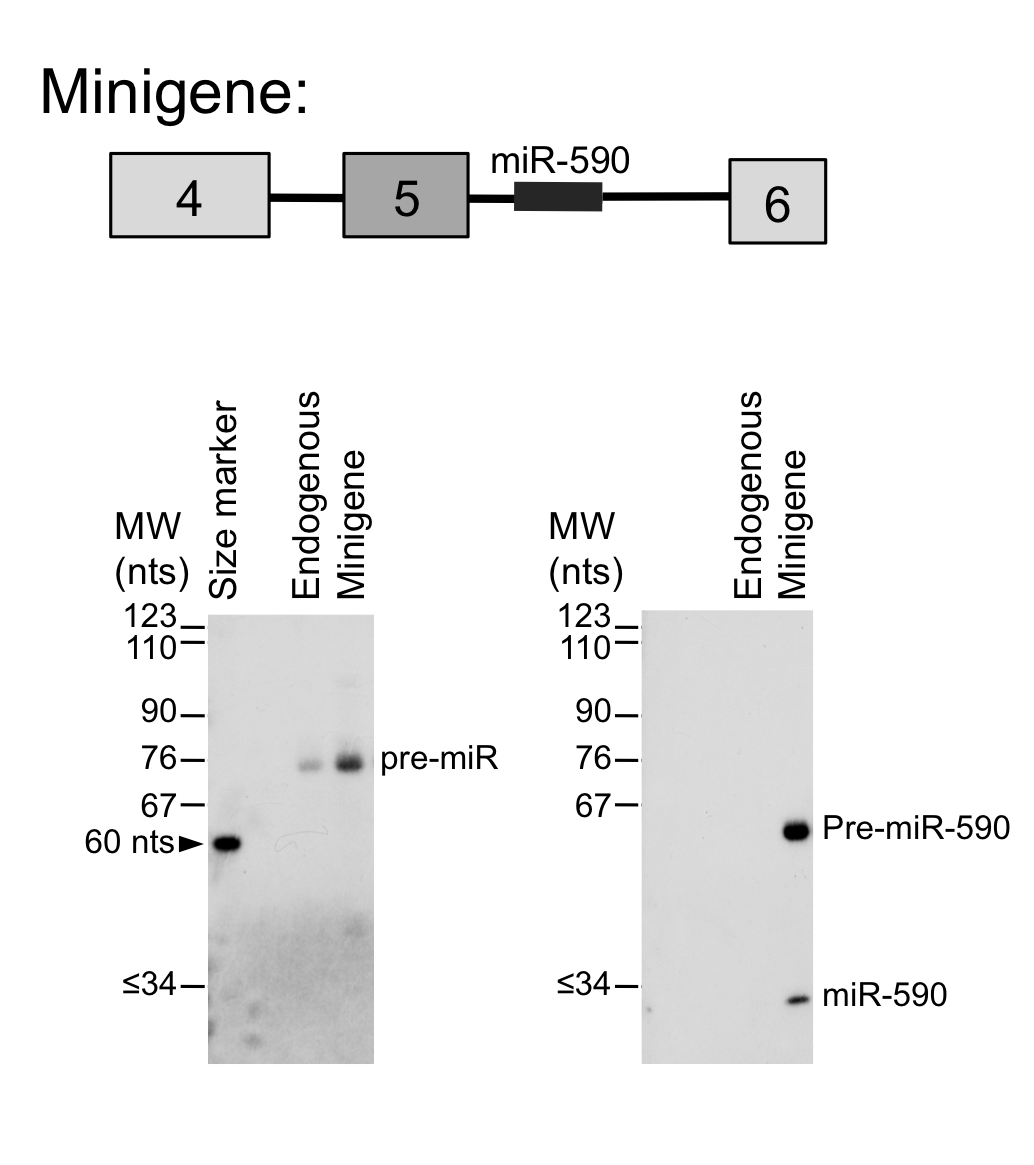

Supplement: Figure S3 — A putative exon 5-derived pre-miRNA is detectable in HeLa cells. Northern blot of endogenous RNA from HeLa cells or HeLa cells transfected with an eIF4H minigene (structure shown in top panel) probed with an LNA probe to exon 5 (left panel), which shows the presence of an excised exon 5 species (pre-miR). Molecular weight markers are shown on the left. A 60 nt size marker consisting of the exon 5 sequence is shown, indicating the size of the exon and the functionality of the LNA probe. Another membrane containing the same samples was probed with an LNA probe to miR-590-5p (right panel). Both pre- and mature miR-590-5p are detected. (TIF) [file pgen.1004312.s003.tif]

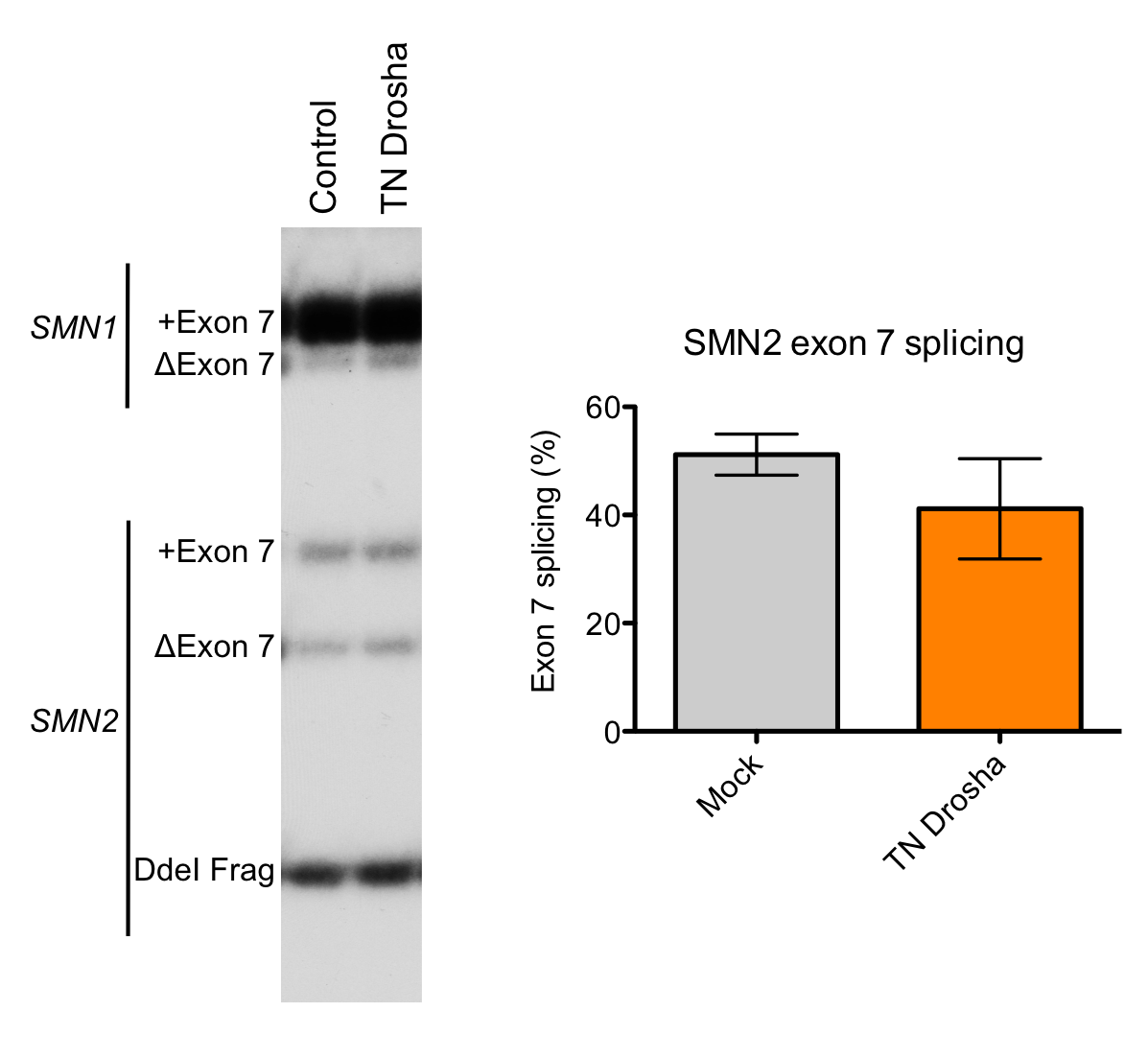

Supplement: Figure S4 — TN Drosha does not affect SMN2 exon 7 splicing. Radiolabelled RT-PCR analysis of endogenous SMN2 exon 7 splicing in control HEK-293T cells or cells that were transiently transfected with TN Drosha. SMN1 and SMN2 were amplified via RT-PCR and digested with DdeI, which only digests SMN2. Graph shows the percent of SMN2 exon 7 splicing [+Exon 7/(+Exon 7+ΔExon 7)*100], n = 3. Error bars represent SEM. (TIF) [file pgen.1004312.s004.tif]

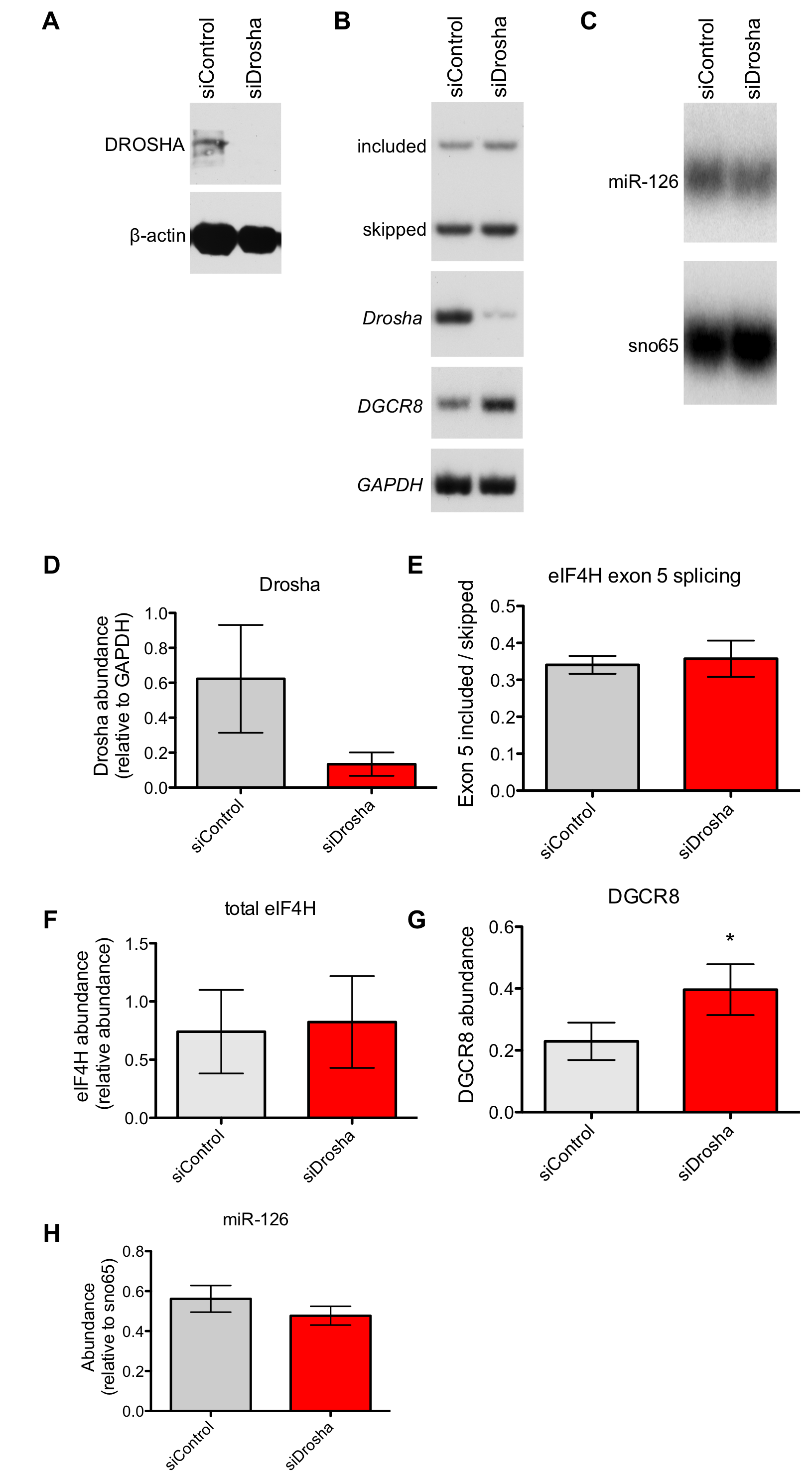

Supplement: Figure S5 — Drosha knockdown does not affect eIF4H exon 5 splicing. (A) An immunoblot of Drosha expression in HeLa cells treated with control siRNA (siControl) or Drosha siRNA (siDrosha). β-actin is a loading control. (B) Radiolabelled RT-PCR analysis of eIF4H exon 5 splicing and expression of Drosha, DGCR8 and GAPDH, a loading control following Drosha RNAi. (C) Radiolabelled stem-loop RT-PCR analysis of a canonical miRNA, miR-126, expression, sno65 is a loading control. Graphs show quantitation of (D) Drosha mRNA levels relative to GAPDH (Drosha/GAPDH), (E) ratio of exon 5 splicing (included/skipped), (F) eIF4H abundance relative to GAPDH [(included+skipped)/GAPDH], (G) DGCR8 expression, a non-canonical target of the Microprocessor, relative to GAPDH (DGCR8/GAPDH) and (H) miR-126 expression relative to sno65 (mir-126/sno65). * indicates statistical significance by Student's t-test where p≤0.05, n = 3. Bars represent SEM. (TIF) [file pgen.1004312.s005.tif]

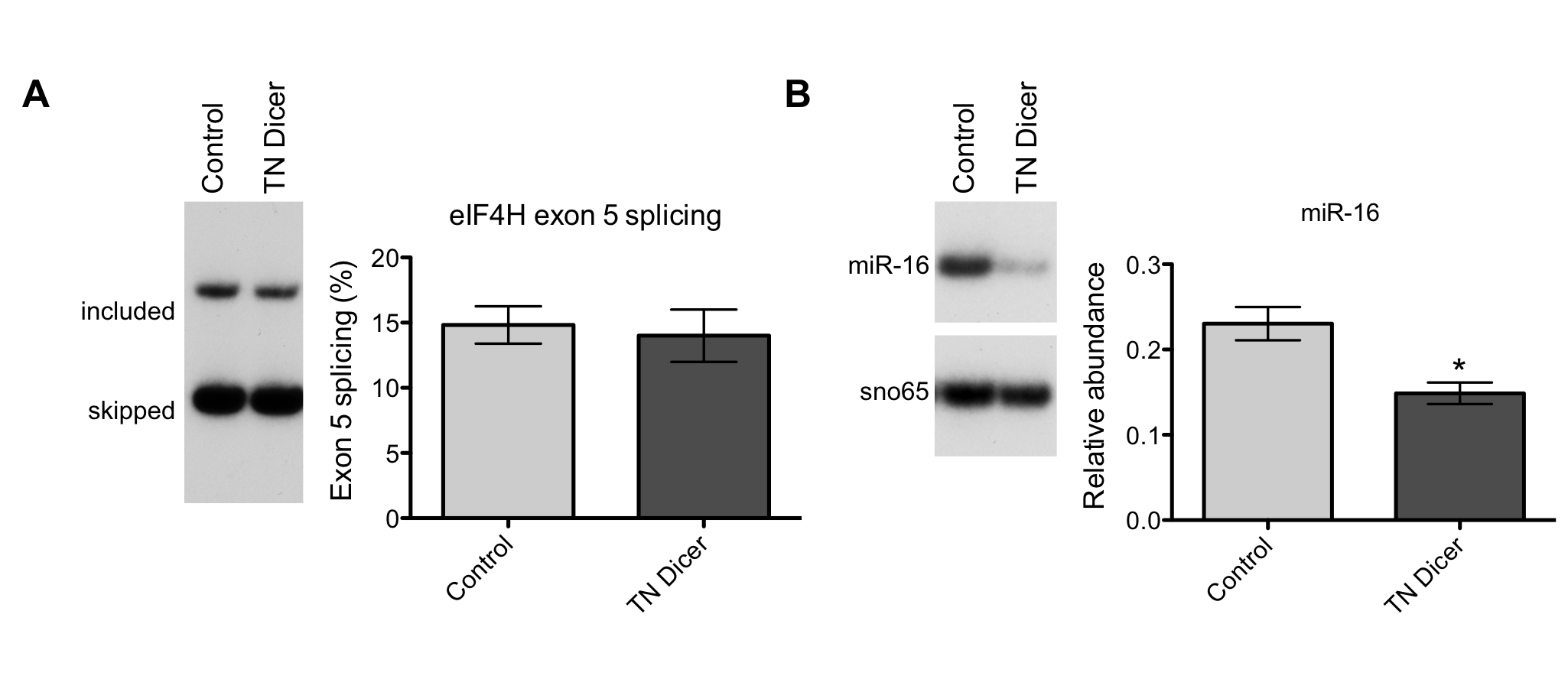

Supplement: Figure S6 — TN Dicer does not affect splicing of eIF4H exon 5. (A) Radiolabelled RT-PCR analysis of eIF4H splicing in control or TN Dicer treated HEK-293T cells. Graph shows percent of exon 5 splicing [included/(included+skipped)*100]. (B) Stem-loop RT-PCR analysis of miR-16 expression in control and TN Dicer treated cells. Graph shows miR-16 expression relative to sno65 (miR-16/sno65). miR-16 is a positive control and sno65 is a loading control. * indicates statistical significance by Student's t-test where p≤0.05, n = 6. Bars represent SEM. (TIF) [file pgen.1004312.s006.tif]

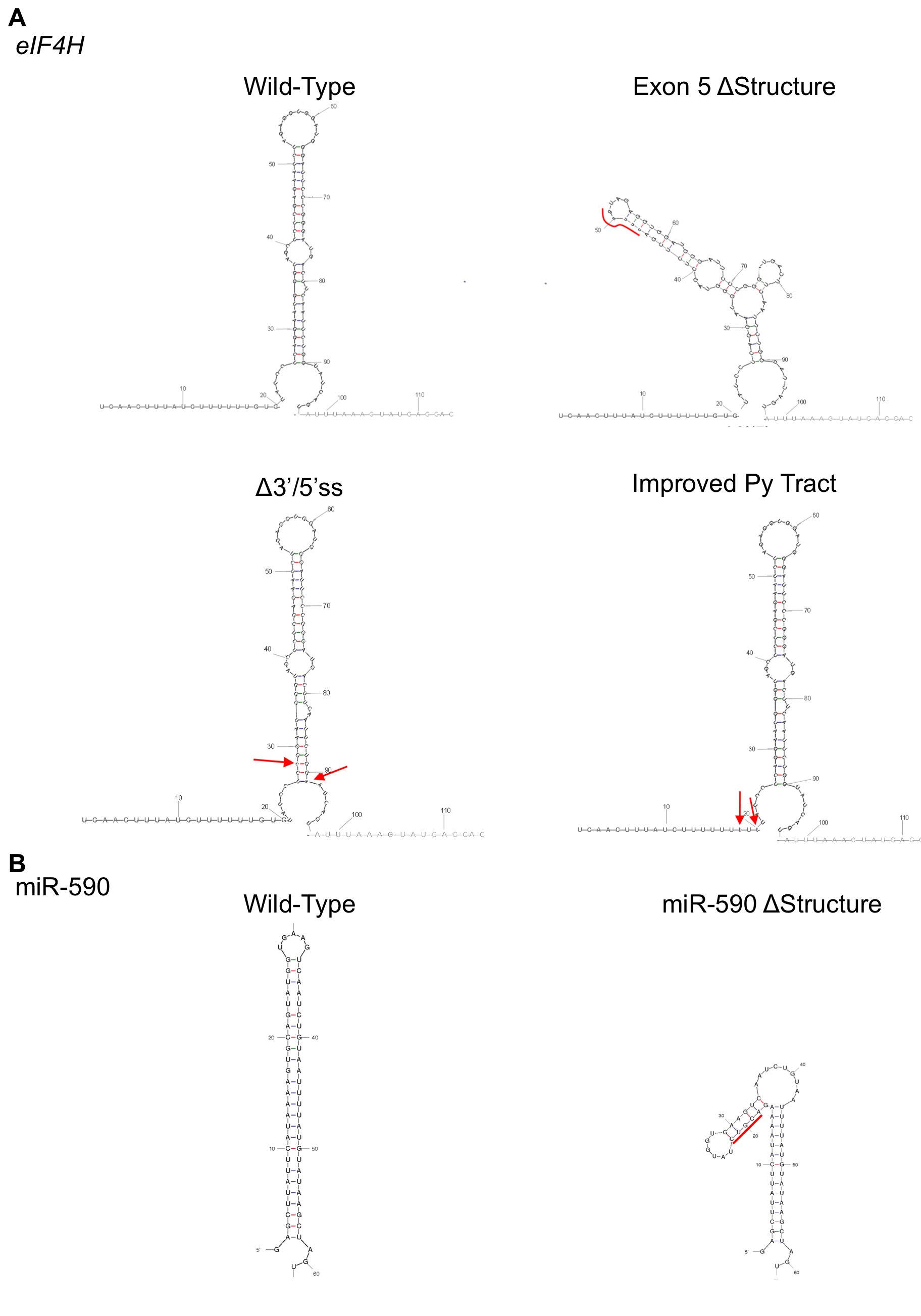

Supplement: Figure S7 — Predicted structures of exon 5 and miR-590 RNA from mutated minigenes. (A) The hairpin structure of eIF4H exon 5 was preserved in all mutated forms of the minigene with the exception of the mutations that intentionally altered the structure of exon 5 (exon 5 ΔStructure) or (B) miR-590 (miR-590 ΔStructure). Each minigene is shown with the exon and flanking intronic sequences. Red arrows and lines indicate the specific mutation for individual minigenes. (TIF) [file pgen.1004312.s007.tif]

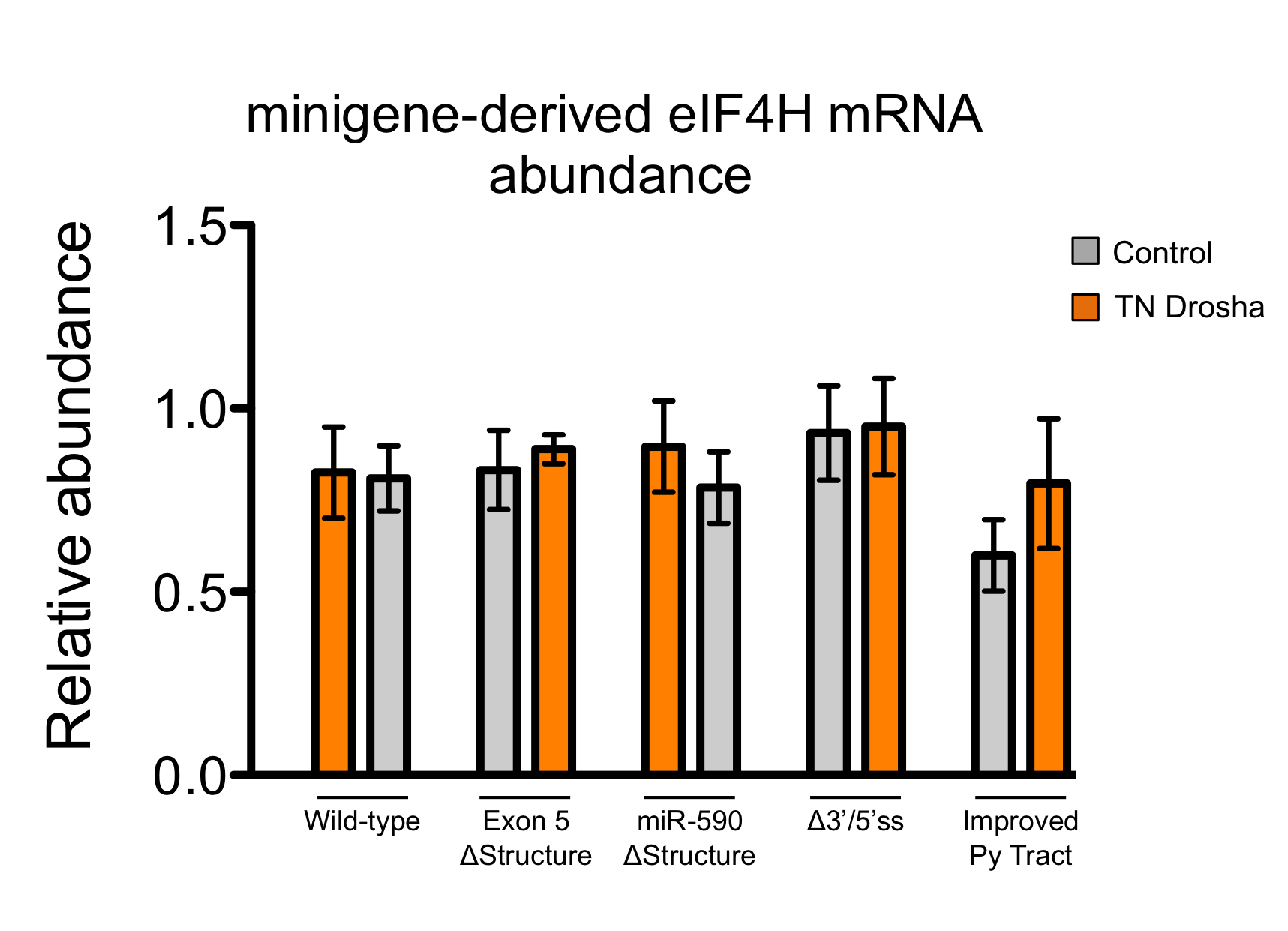

Supplement: Figure S8 — Minigene mutations do not alter eIF4H abundance. The graph indicates overall eIF4H abundance relative to GAPDH in HEK-293T cells transiently transfected with the indicated minigenes and with or without TN Drosha [(included+skipped)/GAPDH]. For minigenes alone, n = 6, for minigenes co-transfected with TN Drosha n = 3. (TIF) [file pgen.1004312.s008.tif]

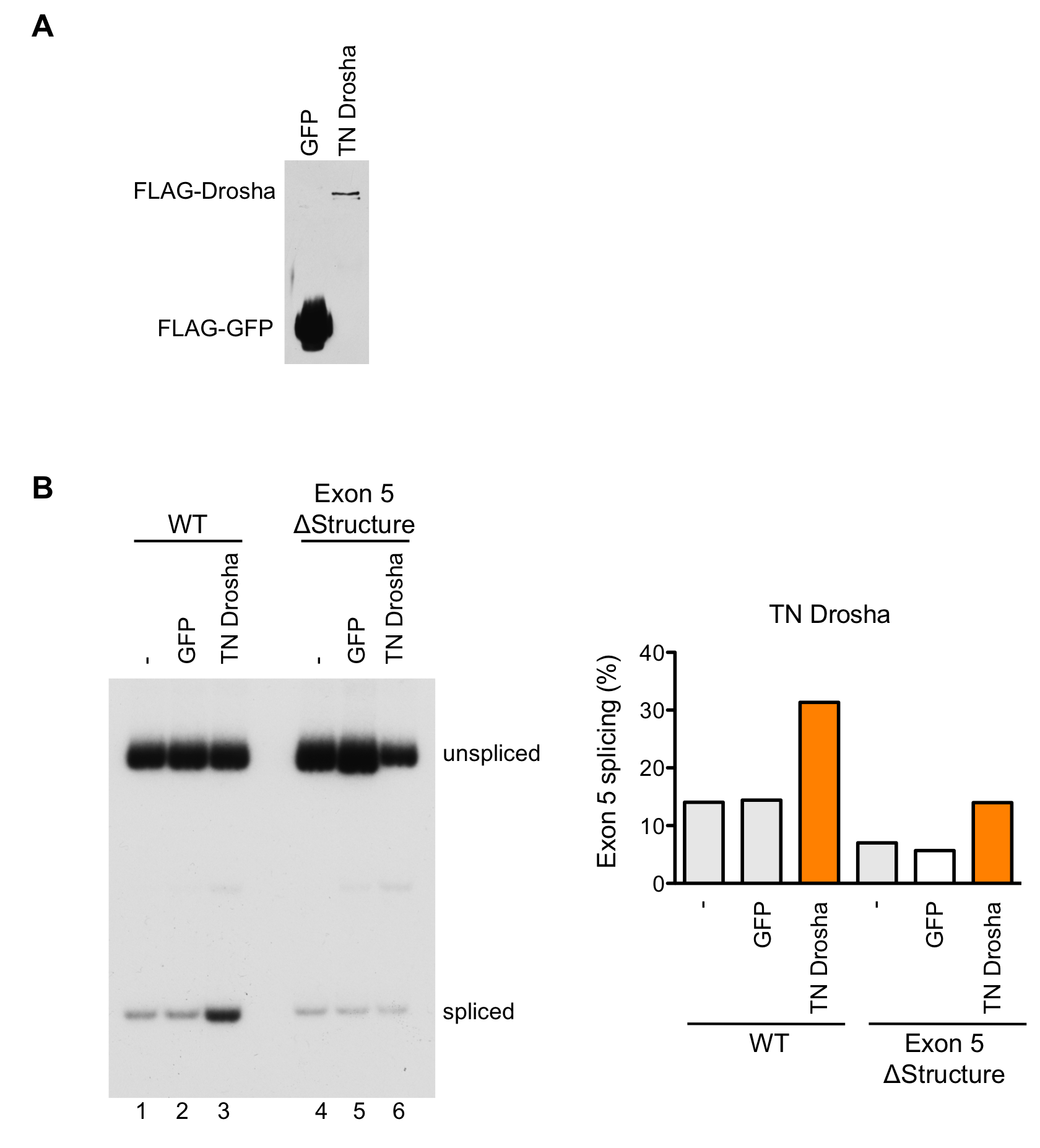

Supplement: Figure S9 — TN Drosha enhances exon 5 splicing in vitro. (A) Immunoblot of FLAG-tagged GFP and TN Drosha probed with anti-FLAG antibody. (B) Radiolabelled RT-PCR analysis of RNA from in vitro splicing reactions of WT and Exon 5 ΔStructure RNA. Splicing reactions were carried out in HeLa nuclear extracts (-, lanes 1 and 4), or nuclear extracts supplemented with FLAG-tagged GFP (lanes 2 and 5), or TN Drosha (lanes 3 and 6). Unspliced and spliced (exon 4 to 5) RNA is indicated. Graph shows the percent of exon 5 splicing [spliced/(spliced+unspliced)*100]. (TIF) [file pgen.1004312.s009.tif]
