# Supplementary material for: Drosha Promotes Splicing of a Pre-microRNA-like Alternative Exon
Source: PLoS Genet. 2014 May 1;10(5):e1004312. doi: 10.1371/journal.pgen.1004312 (PMC4006729; doi:10.1371/journal.pgen.1004312)
Supplement: Table S1 — Primer sequences. Primers used in this study are shown 5′ to 3′. (PDF) [file pgen.1004312.s010.pdf]

Primer Table for eIF4H

	Primer Name	Sequence
**Cloning**	eIF4H exon 4 Forward	CGGCTAGCCTGTTGGGCGATCGGTCACTTCG
	eIF4H exon 6 Reverse	CGGCGGCCGCCTTCTGTGGGTTCTCTG
**in vitro Transcription**		
	eIF4H intron 5 Reverse	CTAAATAAGTAGTCCGGCCAGG
	T7 eIF4H intron 4 Forward	TAATACGACTCACTATAGGCTTCCATCACTTGAGTTGTATG
	T7 SMN1 intron 6 Forward	TAATACGACTCACTATAGGGTCTTGTGAAACAAAATGC
	SMN1 intron 7 Reverse	CCTTTCAACTTTTTAACATC
	T7 APP intron 7 Forward	TAATACGACTCACTATAGGCAGATAGGAAGGGGTATG
	APP Intron 8 Reverse	GTTCATGGGACTATGAAC
	T7 miR-96 Forward	TAATACGACTCACTATAGGCCGGAGCACCTTACCCAC
	miR-96 Reverse	GCGCTGGGCCCTCACAC
	T7 miR-590 Forward	TAATACGACTCACTATAGGCTGGTGCACGCTGTCTTGTG
	miR-590 Reverse	GTAGAAAGACGTTAGTATGTGGGC
	T7 ABCF1 Forward	TAATACGACTCACTATAGGAGGTGGTAGCAGATGAG
	T7 ABCF1 Reverse	ATGGCCCACCTGGCCAGGG
	T7 promoter	TAATACGACTCACTATAGGG
	Exon 5 with ss Reverse	TACTGATACCAGAATTGAAGTC
**QuikChange**	ΔmiR-590 Structure F	GAGCTTATTCATAAAAGACGTCTATGGTGAAGTCAATCTG
	ΔmiR-590 Structure R	CAGATTGACTTCACCATAGACGTCTTTTATGAATAAGCTC
	ΔeIF4H 5' splice site F	GGGATGACTTCAATTCTGGAATCAGTATTTAAAGTATCAC
	ΔeIF4H 5' splice site R	GTGATACTTTAAATACTGATTCCAGAATTGAAGTCATCCC
	eIF4H 3' splice site improved	CGACCTCAACTTTATCTTTTTTTTTTATCCTCAGGAATGGGTAGCTC
	eIF4H 3' splice site improved	GAGCTACCCATTCCTGAGGATAAAAAAAAAAGATAAAGTTGAGGTCG
	Δ eIF4H Exon 5 structure	GTAGCTCTCGACTTAGTAGAGGTGGATG
	Δ eIF4H Exon 5 structure R	CATCCACCTCTACTAAGTCGAGAGCTAC
**Gene specific**	GAPDH Forward	GAAGGTGAAGGTCGGAGTC
	GAPDH Reverse	GAAGATGGTGATGGGATTTC
	eIF4H exon 4 Forward	GGGCGATCGGTCACTTCG
	eIF4H exon 6 Reverse	CGCCTGGGCGACTACCTC
	eIF4H exon 5 Reverse	CAGAATTGAAGTCATCCCGGG
	T7 promoter minigenes	TAATACGACTCACTATAGGG
	pTarget Forward	GGGCGAATTCGGATCCTTG
	5' linker	TGGAATTCTCGGGCACC
	3' linker	ATTGATGGTGCCTAC
	miR-16 Forward	TAGCAGCACGTAAA
	snoRNA65 Forward	TGACTCTGTCCCGAAAGCAT
	Stemloop Reverse	CCAGTGCAGGGTCCGAG
	Drosha Forward	CATGCACCAGATTCTCCTGTA
	Drosha Reverse	GTCTCCTGCATAACTCAACTG
	eIF4H exon 5 Forward	GAATGGGTAGCTCTCGA
	SMN Exon 6 Forward	CGATCTCGAGATAATTCCCCCACCACCTCCC
	SMN Exon 8 Reverse	ATATGCGGCCGCCACATACGCCTCACATACA
	DGCR8 For	TATCAGATCCTCCACGAGTG
	DGCR8 R	TCTTGGAGCTTGCTGAGGAT
	Dicer Forward	GGCCGCCTTTCATATATGAG
	Dicer Reverse	GCCCAGCAGGGCTTCCACACAGTCCGC
	eIF4H Intron 5 R	CTAAATAAGTAGTCCGGCCAGG
	miR-126 For	TCGTACCGTGAGTAA
**Stemloops**	miR-16	GTCGTATCCAGTGCAGGGTCCGAGGTATTCGCACTGGATACGACCGCCAA
	snoRNA65	GTCGTATCCAGTGCAGGGTCCGAGGTATTCGCACTGGATACGACGGCTGT
	miR-126	GTCGTATCCAGTGCAGGGTCCGAGGTATTCGCACTGGATACGACCGCATT
	5p putative Dicer product 1	GTCGTATCCAGTGCAGGGTCCGAGGTATTCGCACTGGATACGACCTACCC
	5p miR	GTCGTATCCAGTGCAGGGTCCGAGGTATTCGCACTGGATACGACAGCTAC
	5p putative Dicer product 3	GTCGTATCCAGTGCAGGGTCCGAGGTATTCGCACTGGATACGACAGATTC
	5p putative Dicer product 4	GTCGTATCCAGTGCAGGGTCCGAGGTATTCGCACTGGATACGACTTCTCG
	5p putative Dicer product 5	GTCGTATCCAGTGCAGGGTCCGAGGTATTCGCACTGGATACGACGATTCT
	3p putative Dicer product 1	GTCGTATCCAGTGCAGGGTCCGAGGTATTCGCACTGGATACGACGAATCC
	3p putative Dicer product 2	GTCGTATCCAGTGCAGGGTCCGAGGTATTCGCACTGGATACGACAATTGA
	3p/pre-miR	GTCGTATCCAGTGCAGGGTCCGAGGTATTCGCACTGGATACGACCCAGAA
	3p putative Dicer product 4	GTCGTATCCAGTGCAGGGTCCGAGGTATTCGCACTGGATACGACATACCA
	3p putative Dicer product 5	GTCGTATCCAGTGCAGGGTCCGAGGTATTCGCACTGGATACGACATCCAC
		**Target sequence**
**Qiagen siRNA**	Drosha 1	ATCGATCAACTGGATCGTGAA
	Drosha 2	TACACGATTCATTTCATTGAA
	Drosha 3	ACGAAGCTCGATGAAGATTTA
	Drosha 4	AAGGGATTAACACCTTGATAA
**IDT RNA linker 1**	Linker 1	rAppCTGTAGGCACCATCAAT/3ddC/
**IDT RNA 5' M.R.S. Linker**	M.R.S. miRNA cloning linker	TGGAATrUrCrUrCrGrGrGrCrArCrCrArArGrGrU
